# Continuous-Time Event-Driven Temporal–Causal Attention for Intention-Driven Anticipation in Badminton

**DOI:** 10.3390/s26196132

**Published:** 2026-09-27

**Authors:** Yiming Gao, Hongzhang Fan, Jinxin Han, Yigu Tian, Jiayi Wang, Zhirong Luan

**Affiliations:** 1 School of Sport and Physical Education, North University of China, No. 3 Xueyuan Road, Shanglan Subdistrict, Taiyuan 030051, China; 2311024108@st.nuc.edu.cn (Y.G.); 2411014230@st.nuc.edu.cn (H.F.); 2311014118@st.nuc.edu.cn (J.H.); 2511024105@st.nuc.edu.cn (Y.T.); 2511014210@st.nuc.edu.cn (J.W.); 2 School of Electrical Engineering, Xi’an University of Technology, Qujiang Campus, No. 58 Yanxiang Road, Xi’an 710054, China

**Keywords:** continuous-time dynamics, neural ordinary differential equations, temporal–causal attention, intention anticipation

## Abstract

High-speed badminton requires defenders to anticipate opponents’ intentions from early kinematic cues under severe temporal and sensory constraints. Existing models rely on discrete-time assumptions and statistical correlations, failing under irregular sampling or missing frames. We propose a Continuous-Time Event-Driven Temporal–Causal Attention (CT-ACI) framework that models the intention state as a neural ordinary differential equation (Neural ODE), embeds a physical causal mask to prevent backward leakage, and introduces an adaptive integral triggering mechanism for event-driven predictions. This framework is designed as a robust anticipation algorithm for irregularly sampled sports visual data, providing early candidate signals for downstream decision support. A curriculum training strategy stabilizes optimization. When evaluated on the VideoBadminton dataset, the framework maintains stable prediction under severe missing frame conditions where discrete baselines degrade substantially. In addition, it reduces the long-term anticipation error while providing a binary intention signal under strict temporal–causal constraints. The trigger time $T^{*}$ is consistently treated as an operational proxy derived from observable defender motion, not as a validated cognitive decision boundary. The dataset contains only six singles matches and no player identifiers, while two matches are shared between training and test; thus, the reported results characterize generalization to unseen rallies and clips rather than to unseen matches or players.

## 1. Introduction

In high-speed racquet sports such as badminton, the ability to anticipate an opponent’s intention from early kinematic cues is essential for both real-time decision-making and post-match tactical analysis [1]. Automatically extracting such cues from raw match footage is a compelling yet challenging goal because the relevant signals are subtle, embedded in highly dynamic scenes, and often obscured by motion blur, occlusion, and irregular skeleton sampling. These difficulties motivate our central problem: inferring intention from irregular partially-observed action sequences under temporal–causal constraints.

Existing intention anticipation models, ranging from template matching [2,3] to recurrent networks, transformers, and graph convolutions [4,5,6,7,8,9,10,11], share a common assumption that they operate on discrete equally-spaced time steps. This synchronization with a fixed temporal grid enables high precision on uniformly sampled sequences, but becomes a critical liability when the grid breaks under the conditions of motion blur, self-occlusion, and camera switches common in real matches. Forced interpolation or padding disrupts the physical continuity of human motion [12,13] and introduces cumulative feature propagation errors. Beyond discrete sampling, most models lack explicit temporal causal constraints, leaving them vulnerable to spurious correlations and temporal data leakage. Furthermore, they produce predictions at fixed frame rates, ignoring the inherently event-driven nature of human decision-making. A defender typically reacts when accumulated evidence reaches a cognitive threshold [14,15], yet this continuous accumulation process remains unmodeled in existing approaches.

We propose a continuous-time event-driven temporal–causal attention framework, called CT-ACI. It models the opponent’s intention as a continuous dynamical system via neural ordinary differential equations (neural ODEs) [16] in order to naturally handle irregular sampling without interpolation. A strict physical causal mask eliminates backward information leakage and an adaptive integral triggering mechanism converts the accumulated causal evidence into an event-driven decision, reflecting human visuomotor delay. A curriculum-based training strategy stabilizes the joint optimization.

Throughout this paper, the terms “causal” and “temporal–causal attention” refer to temporal non-anticipativity rather than statistical or interventional causality. Specifically, predictions at time *t* are constrained to depend only on observations acquired at or before *t*, which is enforced by the temporal–causal attention mask described in Section 3.4. The proposed framework does not estimate causal effects, construct structural causal models, or perform counterfactual inference in the Pearlian sense [17]. Thus, the term “causal” denotes chronological information flow under an online prediction constraint.

The main contributions of this work are threefold:

1. We propose the Continuous-Time Event-Driven Temporal-Causal Attention (CT-ACI) framework for intention anticipation from irregularly sampled sports visual data. The framework models the opponent’s intention state with neural ODEs, enabling natural handling of non-uniform observations with intrinsic robustness to missing frames.
2. We introduce a strict physical temporal–causal mask to eliminate backward information leakage and an adaptive integral triggering mechanism that converts anticipation into an event-driven decision driven by accumulated causal evidence.
3. The proposed framework maintains stable performance under severe missing frame conditions and substantially reduces long term anticipation error compared to discrete baselines.

This work is aligned with the theme of this Special Issue on advanced sensing technologies in sports biomechanics, as it addresses practical sensor-level challenges that arise in visual sensing pipelines for badminton. Video-derived pose streams are often irregularly timestamped because frame drops, decoding jitter, and variable processing delays induce non-uniform inter-frame intervals. CT-ACI’s neural ODE backbone integrates over these variable intervals directly, removing the need for resampling or interpolation that can distort motion timing. Skeleton extraction under occlusion or motion blur produces intermittent missing detections; the temporal–causal attention mechanism allows the model to update only when new observations arrive without requiring a fixed frame rate. The event-driven threshold trigger decouples prediction timing from the input frame rate, so outputs can be emitted when accumulated evidence suffices rather than at fixed intervals. These properties suggest that CT-ACI can serve as a flexible inference component for visual sensor pipelines that must operate under realistic bandwidth, latency, and packet loss constraints. While the VideoBadminton dataset does not provide camera-specific calibration or noise models, the Data Acquisition and Preprocessing subsection (Section 3.1) fully specifies the extraction pipeline, frame rates, and handling of low-confidence or missing observations.

## 2. Related Work

### 2.1. Action Anticipation and Discrete Temporal Modeling

Action anticipation requires inferring future states from early spatiotemporal cues before action completion [18,19,20]. Early work used template matching and hidden Markov models (HMMs) [21,22], but was limited by the need for hand-crafted features. Deep learning advanced the field with two-stream networks, three-dimensional convolutional neural networks (3D CNNs) such as Inflated 3D ConvNet (I3D) and SlowFast [23,24], and spatial–temporal graph convolutional networks (ST-GCNs) [25,26]. These models learn spatiotemporal representations and achieve strong performance on benchmarks.

Nevertheless, both classical and deep models share the fundamental assumptions that input sequences are uniformly sampled on the time axis and that hidden states update at fixed steps. In real badminton matches, fast limb movements cause motion blur, while self-occlusion and camera switches lead to irregular skeleton sequences [27] with missing frames or misaligned timestamps. Faced with irregular sampling, existing methods resort to linear interpolation or zero padding [28], which breaks physical continuity and introduces cumulative errors.

### 2.2. Temporal–Causal Attention in Video Understanding

Purely data-driven anticipation models fit statistical correlations without enforcing temporal causal order, making them prone to spurious correlations (e.g., court background) and weak generalization. Temporal–causal attention theory has been introduced to address this limitation [29]. This work is directly relevant because it disentangles spatial and temporal graph convolutions for skeleton-based action recognition, showing that separating temporal and spatial information reduces entangled spurious spatiotemporal dependencies; this is precisely the issue that our temporal–causal attention mask addresses by constraining information flow along the time axis. Researchers have employed structural causal models and counterfactual interventions [17] to disentangle environment from core features. However, most existing work on causal video understanding remains limited to discrete time slices or strictly aligned sequences [29,30]. In real sports competitions, the propagation of micro-kinematic cues and the triggering of cognitive thresholds form a continuous event-driven process with notable delay effects. Existing methods lack continuous-time causal modeling and cannot enforce strict non-anticipativity under non-uniform sampling or dynamic delays.

### 2.3. Continuous-Time Dynamics and Neural ODEs

Neural ODEs and their variants provide a mathematical framework for handling non-uniform time series by representing hidden states as continuous differentiable functions [31,32]. They allow for exact integration at arbitrary time points using ODE solvers, naturally accommodating irregularly sampled observations without heuristic interpolation. Recent studies have demonstrated their potential for processing non-uniform video and trajectory data. Nevertheless, these continuous-time models have been applied mainly to macroscopic trajectory extrapolation or pixel interpolation; they do not address micro-kinematic evolution driven by internal cognition, and do not model cognitive delays or physiological thresholds as differentiable dynamic conditions. No prior work has unified continuous-time dynamics, physical causal constraints, and cognitive threshold triggering for high-speed sports intention anticipation.

## 3. Materials and Methods

We propose a CT-ACI framework for intention anticipation in high-speed badminton. The framework integrates three complementary mechanisms that work in concert. At its core, the opponent’s intention state is modeled as a continuous dynamical system governed by neural ODEs. This continuous representation provides a backbone that naturally handles non-uniformly sampled observations without heuristic interpolation. Building on this backbone, a strict physical temporal causal mask is embedded in a causal attention mechanism to constrain how information flows, thereby eliminating backward leakage and ensuring that only past observations contribute to predictions. Finally, a context-aware adaptive integral triggering condition converts the accumulated causal evidence into an event-driven decision by modeling the effect of human cognitive delay as a differentiable dynamic threshold. The overall architecture is illustrated in Figure 1.

### 3.1. Data Acquisition and Preprocessing

All experiments are conducted on the publicly available VideoBadminton dataset [33], which provides RGB footage and filename-based frame coordinates. The dataset does not disclose camera models or calibration parameters; consequently, no calibrated 3D reconstruction or per-joint pose error statistics (e.g., MPJPE) can be computed. A source audit found 30 FPS for nearly all parsed matches (one exception at 23 FPS), with timestamps derived from decoded frame indices and reported frame rates.

Skeleton extraction was performed using MediaPipe Pose Landmarker (version 2.0) [34], which detects 25 2D landmarks per person.

Several considerations informed the choice of MediaPipe. A detailed comparison of representative 2D pose estimation frameworks, including their accuracy–speed trade-offs under a unified evaluation protocol, is provided in Section 4.8. Accuracy is assessed via the hip-center localization error relative to the ViTPose-B reference on a stratified subset of 100 test clips, where a lower error indicates higher accuracy. ViTPose-B (COCO checkpoint, ViTPose repo v1.0) is used as a practical reference, not as ground truth, since it achieves the highest reported COCO AP among the compared frameworks. A detailed explanation of the reference selection is provided in Section 4.8. ViTPose-B achieves the highest accuracy, but requires GPU acceleration and substantially higher computational cost; RTMPose-m (MMPose v1.3.0) achieves competitive accuracy with a CPU-friendly inference, while MediaPipe Pose Landmarker (v0.10.x, full model) provides the fastest CPU inference at 15–25 ms per frame and requires no specialized hardware. For the present dataset, the primary constraints are large-scale batch processing over thousands of video clips and the absence of specialized GPU hardware. Under these constraints, MediaPipe offers the most favorable balance between accuracy and throughput. Although RTMPose-m achieves lower hip-center error at comparable CPU speed, MediaPipe provides a more mature cross-platform ecosystem and tighter integration with downstream skeleton processing pipelines. The cross-estimator consistency analysis in Section 4.8 further confirms that the derived $T^{*}$ labels are highly consistent across all compared estimators, indicating that the choice of MediaPipe does not introduce systematic bias into the target definition.

For each detected individual, the pipeline stores normalized (x,y) coordinates and confidence scores (a 75-dimensional pose vector). Low-confidence joints are retained in the pose vector without filtering so that the neural ODE can integrate over intermittent missing detections. For the specific computation of defender hip-center velocity and $T^{*}$, frames with hip confidence below 0.5 are treated as missing observations. The primary player (attacker) is identified by landmark count and body extent; the remaining individual, if present, provides the defender stream. For the hip landmarks, which are directly used to compute defender velocity and $T^{*}$, the hip-center position is computed as the midpoint between the two hip landmarks; the hip confidence is defined as the minimum of the confidence scores of these two landmarks, following a conservative criterion that a frame is considered reliable only when both hip landmarks are reliably detected. The failure threshold is set to 0.5, consistent with the low-confidence criterion used to handle missing frames throughout the pipeline.

Trigger-time labels are deterministically derived from defender hip-center speed computed in the normalized image coordinate space. Since the dataset provides no camera calibration or scale recovery, all velocity quantities are dimensionless scalars. We denote them in normalized velocity units (NVU) throughout the manuscript, calculated as the Euclidean distance between consecutive hip-center positions divided by the inter-frame interval. Specifically, $T^{*}$ is defined as the timestamp immediately following the first smoothed speed sample satisfying $v_{\mathrm{smooth}}\geq 0.3$ (NVU), with fallback thresholds of 0.1 (NVU) or the peak-speed timestamp if no crossing occurs. Among all clips, 6847 (87.5%) use the primary 0.3 threshold, 612 (7.8%) use the 0.1 fallback, and 364 (4.7%) use the peak-speed timestamp; this distribution is reported here in order to ensure full transparency in the label generation process. $T^{*}$ is an operationally defined proxy for the onset of observable defender motion, not a direct measurement of a physiological or cognitive decision boundary. The future defender trajectory is linearly interpolated over a 1.5-s horizon (151 discrete points). ADE and FDE are reported in normalized image coordinate units (multiplying the raw normalized errors by 100 for numerical convenience). Because the dataset provides no camera calibration, homography, or metric reconstruction, these values are not calibrated physical distances and should not be interpreted in centimeters; the numerical magnitudes are meaningful only for relative comparisons between methods evaluated on the same test data. The preprocessing pipeline is deterministic and fully scripted; code and feature files are available upon request.

When skeleton extraction fails for a frame (e.g., due to occlusion or motion blur), the frame is treated as a missing observation rather than being interpolated or padded. The neural ODE solver then integrates over the resulting temporal gap, as described in Section 3.3.

### 3.2. Problem Definition

Let the continuous time axis be $t\in R_{\geq 0}$. Due to sensor sampling limits, motion blur, and occlusion, the opponent’s kinematic observations are non-uniform in time:

(1) $$O={\left\{\left(\tau_{i},o_{i}\right)\right\}}_{i=1}^{N},\;0\leq \tau_{1}<\tau_{2}<\ldots <\tau_{N}$$

where $\tau_{i}$ is the observation time and $o_{i}\in R^{D_{o}}$ is the corresponding kinematic feature vector. The inter-observation interval $\Delta_{i}=\tau_{i}-\tau_{i-1}$ is non-constant, reflecting missing frames and irregular sampling.

Let $z\left(t\right)\in R^{D_{z}}$ denote the model’s continuous estimate of the opponent’s latent intention state, inferred from the kinematic observation sequence. Let $r\left(t\right)\in R^{D_{r}}$ denote the defender state. Before the anticipatory response, this state encodes observable preparation cues such as stance and racket position; after the response, it serves as the target of trajectory prediction. The defender’s reaction is triggered when the accumulated temporally ordered evidence first exceeds a context-dependent dynamic threshold. Formally, the accumulated evidence is given by $\int_{0}^{t}{∥z(s)∥}_{2}ds$, and the threshold θ(r(t)) is parameterized by the defender’s pre-trigger state. We define an operational trigger proxy *T* as the earliest time at which the accumulated evidence reaches the adaptive threshold

(2) $$T=\inf\left\{t>0:\int_{0}^{t}{∥z\left(s\right)∥}_{2}ds\geq \theta \left(r\left(t\right)\right)\right\},$$

where θ(r(t)) is parameterized as described in Section 3.5. This proxy is derived from observable defender motion, and is not claimed to represent a physiological or cognitive decision boundary.

For any time t>T, the defender’s motion r(t) is the future trajectory to be predicted. The available information up to a cutoff time $t_{obs}$ consists of both the opponent’s observation subset $O_{<t_{obs}}=\left\{\left(\tau_{i},o_{i}\right):\tau_{i}<t_{obs}\right\}$ and the defender’s observed kinematic history r(t) for $t\leq t_{obs}$. The core task is to learn a mapping that predicts the triggering time $\hat{T}$ and the subsequent defender trajectory $\hat{r}\left(t\right)$ for $t>\hat{T}$, minimizing the prediction error.

### 3.3. Continuous Intention Dynamics with Neural ODEs

Traditional discrete sequence models assume state updates of the form $z_{k}=z_{k-1}+\Delta t\cdot f\left(z_{k-1},o_{k}\right)$ with a fixed step size. When actual observation intervals are non-constant or many frames are missing, this forced discretization leads to either discarding non-aligned observations or introducing spurious correlations via interpolation. Therefore, we adopt a continuous perspective.

We model the intention state evolution as an initial value problem:

(3) $$\frac{dz(t)}{dt}=f_{\phi }\left(z\left(t\right),t\right),\;z\left(0\right)=z_{0}$$

where $f_{\phi }:R^{D_{z}}\times R\to R^{D_{z}}$ is a neural network with learnable parameters ϕ that parameterizes the time derivative. The solution is given by $z\left(t\right)=z\left(0\right)+\int_{0}^{t}f_{\phi }\left(z\left(s\right),s\right)ds$, computed numerically by an ODE solver. In this work, we use the Dormand–Prince method of order 5 (Dopri5) solver, which adaptively adjusts step sizes to control local truncation error. The key mathematical advantage is that non-uniform observation times $\tau_{i}$ act solely as query points for the solver output, not as step sizes; consequently, even when a long gap exists between two observations, the model maintains a continuous state evolution by integrating $f_{\phi }$, requiring no interpolation.

To incorporate the discrete observations into the continuous dynamics, we first compute an initial intention state from the earliest available window of observations. A lightweight temporal encoder $\phi_{enc}$ (e.g., a causal one-dimensional (1D) convolution or a short transformer) takes a small number of observed frames and outputs the initial hidden state z(0). The continuous dynamics then evolve from this initial state according to the neural ODE:

(4) $$\begin{array}{cc} \frac{dz(t)}{dt} & =f_{\phi }\left(z\left(t\right),t\right),\\ z(0) & =\phi_{enc}\left({\left\{o_{i}\right\}}_{i=1}^{k}\right). \end{array}$$

At each observation time $\tau_{i}$, the continuously-evolving intention state $z\left(\tau_{i}^{-}\right)$ (the state immediately before the update) is corrected by incorporating the current kinematic observation $o_{i}$ through a temporal–causal attention mechanism (detailed in Section 3.4). This yields a posterior update

(5) $$z\left(\tau_{i}^{+}\right)=z\left(\tau_{i}^{-}\right)+h_{i},$$

where $h_{i}$ is the attention output computed from the history of pre-update states and the current observation. The updated state $z\left(\tau_{i}^{+}\right)$ then serves as the new initial condition for the ODE solver in the subsequent interval $\left[\tau_{i},\tau_{i+1}\right)$. This design naturally accommodates non-uniform observation times, as the intention state evolves according to the learned vector field $f_{\phi }$ between observations and is corrected by incoming evidence at each observation time.

For end-to-end training, we employ the adjoint sensitivity method. This method computes gradients of the loss with respect to $z_{0}$ and ϕ by solving an augmented adjoint differential equation backward in time, without storing intermediate states of the forward integration. Specifically, define the adjoint state a(t)=∂L/∂z(t), where L is the total loss; then, the adjoint satisfies

(6) $$\frac{da(t)}{dt}=-a{\left(t\right)}^{⊤}\frac{\partial f_{\phi }\left(z\left(t\right),t\right)}{\partial z}.$$

At each observation time $\tau_{i}$, it experiences a posterior condition that reflects the gradient of the attention update. Let $a(\tau_{i}^{-})$ and $a(\tau_{i}^{+})$ denote the adjoint state immediately before and after $\tau_{i}$. The posterior condition is

(7) $$a\left(\tau_{i}^{-}\right)=a\left(\tau_{i}^{+}\right)+{\left(\frac{\partial h_{i}}{\partial z(\tau_{i}^{-})}\right)}^{⊤}a\left(\tau_{i}^{+}\right),$$

where $\frac{\partial h_{i}}{\partial z(\tau_{i}^{-})}$ is the Jacobian of the attention output with respect to the pre-update state.

Importantly, this posterior only accounts for the direct modification at $\tau_{i}$. The gradients with respect to earlier states are automatically propagated by the adjoint differential equation backward in time. Thanks to the adjoint method, the memory complexity of the ODE integration is independent of the number of solver steps K, i.e., $O(D_{z}+D_{\phi })$.

### 3.4. Temporal–Causal Attention with Physical Temporal–Causal Mask

At each observation time $\tau_{i}$, we have the pre-update ODE state $z(\tau_{i}^{-})$, which is obtained by integrating from the previous posterior point. To incorporate the current observation $o_{i}$ while strictly preventing any backward information leakage, we construct the update via a temporal–causal attention mechanism that attends over all past pre-update states and also injects the current input.

Formally, let the queries, keys, and values be linear projections of the history of pre-update states ${\left\{z(\tau_{j}^{-})\right\}}_{j=1}^{i}$:

(8) $$\begin{array}{cc} q_{i} & =W_{Q}z\left(\tau_{i}^{-}\right),\\ k_{j} & =W_{K}z\left(\tau_{j}^{-}\right),\\ v_{j} & =W_{V}z\left(\tau_{j}^{-}\right), \end{array}$$

where $W_{Q},W_{K},W_{V}$ are learnable matrices. The attention weight from target time $\tau_{i}$ to past time $\tau_{j}$ is defined as

(9) $$\alpha_{ij}=\frac{\exp\left(\frac{q_{i}^{⊤}k_{j}}{\sqrt{d}}+m_{ij}\right)}{\sum_{k=1}^{i}\exp\left(\frac{q_{i}^{⊤}k_{k}}{\sqrt{d}}+m_{ik}\right)},$$

with the strict physical temporal–causal mask

(10) $$m_{ij}=\left\{\begin{array}{cc} 0, & \mathrm{if}\tau_{j}<\tau_{i},\\ -\infty , & \mathrm{otherwise}, \end{array}\right.$$

where d is the dimension of the query/key vectors. This temporal–causal mask guarantees that for any target time $\tau_{i}$, only observations from strictly earlier times contribute, thereby blocking any backward information flow.

The historical context vector is then computed as ${\tilde{h}}_{i}=\sum_{j=1}^{i}\alpha_{ij}v_{j}$. The final attention output that drives the posterior update is obtained by fusing this context with the current observation:

(11) $$h_{i}=\sigma \left(W_{o}{\tilde{h}}_{i}+W_{x}o_{i}\right)$$

where $W_{o}$ and $W_{x}$ are learnable projection matrices and σ is an activation function (e.g., tanh). The updated state is then $z\left(\tau_{i}^{+}\right)=z\left(\tau_{i}^{-}\right)+h_{i}$. This update rule ensures that at each observation time, the model explicitly conditions on both past intention dynamics and the newly arrived kinematic evidence.

In backpropagation, the adjoint equations include a corresponding posterior condition derived from the attention output. Since the state is modified as $z\left(\tau_{i}^{+}\right)=z\left(\tau_{i}^{-}\right)+h_{i}$, the adjoint state must satisfy

(12) $$a\left(\tau_{i}^{-}\right)=a\left(\tau_{i}^{+}\right)+{\left(\frac{\partial h_{i}}{\partial z(\tau_{i}^{-})}\right)}^{⊤}a\left(\tau_{i}^{+}\right),$$

where $\frac{\partial h_{i}}{\partial z(\tau_{i}^{-})}$ is the Jacobian of the attention output with respect to the pre-update ODE state. This term propagates gradients through the attention mechanism and the observation fusion back into the continuous dynamics. Note that the gradients of $h_{i}$ with respect to earlier states $z\left(\tau_{i}^{-}\right)\left(j<i\right)$ are not enforced by the posterior condition at $\tau_{i}$ but are instead naturally carried by the continuous adjoint dynamics backward from $\tau_{i}$ to earlier times, since $h_{i}$ influences the initial condition of the ODE segment after $\tau_{i}$. This ensures stable gradient flow across the continuous-discrete interface.

### 3.5. Adaptive Integral Triggering and Curriculum Optimization

We define the accumulated temporal–causal evidence as the $L_{2}$ integral of the intention state: $\Psi \left(t\right)=\int_{0}^{t}{∥z(s)∥}_{2}ds$. The defender’s cognitive threshold is not fixed but adapts to his own kinematic state r(t). The defender state r(t) in the pre-trigger phase is directly obtained from the defender’s observed skeleton sequence, which is available alongside the opponent’s data in our setup. In practice, we use a short temporal window of defender poses to extract a compact feature vector that conditions the threshold. We model the adaptive threshold as

(13) $$\theta \left(t\right)=\sigma \left(W_{\theta }r\left(t\right)+b_{\theta }\right)\cdot \left(\theta_{\max}-\theta_{\min}\right)+\theta_{\min},$$

where σ is the sigmoid function, $W_{\theta }$ and $b_{\theta }$ are learnable parameters, and $\theta_{\max},\theta_{\min}$ are pre-defined physiological bounds that constrain the threshold to a biologically plausible range (e.g., the minimal and maximal reaction times converted to equivalent evidence levels).

In typical badminton rallies, the defender’s preparation posture changes smoothly within the short anticipation window, usually less than 1s. The accumulated evidence indicates that $\Psi \left(t\right)=\int_{0}^{t}{∥z\left(s\right)∥}_{2}ds$ is non-decreasing and becomes strictly increasing whenever ${∥z\left(s\right)∥}_{2}>0$. The adaptive threshold θ(t) is bounded by construction and varies smoothly with the observed defender state. Therefore, we define the event time as the earliest threshold crossing T=inf{t>0:Ψ(t)≥θ(t)} rather than assuming a unique crossing for all possible sequences. If the evidence signal fluctuates near the threshold, later re-crossings do not alter the definition of *T*, and the soft relaxation described below provides a differentiable training surrogate. The curriculum learning strategy further reduces early-stage instability by progressively introducing difficult sparse observation samples. The triggering condition is the first time when the accumulated evidence meets or exceeds the threshold Ψ(t)≥θ(t).

To make this discrete event differentiable for end-to-end training, we introduce a sigmoid relaxation of the triggering condition and define a soft trigger probability

(14) $$p_{\mathrm{trig}}\left(t\right)=\sigma \left(\beta \left(\Psi (t)-\theta (t)\right)\right),$$

where β>0 is a sharpness parameter. As β→∞, the function converges to the step function; in practice, we set β large enough to approximate hard triggering while maintaining non-zero gradients near the threshold. During inference and for the reported TTE metric, the predicted triggering time is obtained by the hard first-crossing rule

(15) $$\hat{T}=\min\left\{\tau_{i}:p_{\mathrm{trig}}\left(\tau_{i}\right)>0.5\right\},$$

while the differentiable relaxation is used only for training.

During training, the hard event time $\hat{T}$ is replaced by a differentiable soft event time $\tilde{T}$:

(16) $$\rho_{i}=p_{\mathrm{trig}}\left(\tau_{i}\right)\prod_{j<i}\left(1-p_{\mathrm{trig}}\left(\tau_{j}\right)\right),\;\omega_{i}=\frac{\rho_{i}}{\sum_{k=1}^{N}\rho_{k}+\epsilon },\;\tilde{T}=\sum_{i=1}^{N}\omega_{i}\tau_{i},$$

where ϵ is a small numerical constant that prevents division by zero. The weights $\omega_{i}$ define a soft first-crossing distribution over the observed time steps, with larger weights assigned near the earliest threshold crossing. Therefore, the time prediction loss is computed on the differentiable surrogate:

(17) $$L_{\mathrm{time}}={\left(\tilde{T}-T^{*}\right)}^{2}.$$

This formulation allows gradients to flow through $\omega_{i}$, $p_{\mathrm{trig}}\left(\tau_{i}\right)$, Ψ(t), and θ(t), then back to the neural ODE state z(t) and the threshold parameters $W_{\theta },b_{\theta }$.

To encourage the relaxed trigger to remain consistent with the reference event boundary, we further add a threshold-ordering regularization term:

(18) $$\begin{array}{cc} L_{\mathrm{reg}}= & \int_{0}^{T^{*}}\max{\left(0,\Psi (t)-\theta (t)\right)}^{2}dt\\ \; & +\int_{T^{*}}^{\infty }\max{\left(0,\theta (t)-\Psi (t)\right)}^{2}dt. \end{array}$$

This term penalizes premature threshold crossing before $T^{*}$ and insufficient accumulated evidence after $T^{*}$. During inference and for the reported TTE metric, we use the hard stopping rule defined in Equation (15).

In addition, we have a classification loss $L_{class}$ for intention category and a trajectory prediction loss $L_{traj}$ for the defender’s subsequent motion. The full loss is

(19) $$L=\lambda_{1}L_{time}+\lambda_{2}L_{reg}+L_{class}+L_{traj},$$

where $\lambda_{1}$ and $\lambda_{2}$ are hyperparameters that balance the auxiliary terms against the primary losses, $L_{class}$ is the cross-entropy loss over the binary intention labels, namely, aggressive (clear/smash) versus soft (drop), defined as $L_{class}=-\frac{1}{M}\sum_{i=1}^{M}\left[y_{i}\log{\hat{y}}_{i}+\left(1-y_{i}\right)\log\left(1-{\hat{y}}_{i}\right)\right]$, and $L_{traj}$ is the mean squared error (MSE) between the predicted and ground truth defender trajectories over a fixed horizon after the predicted trigger time, defined as $L_{traj}=\frac{1}{L}\sum_{k=1}^{L}{∥\hat{r}\left(t_{k}\right)-r\left(t_{k}\right)∥}_{2}^{2}$.

The VideoBadminton dataset provides three fine-grained stroke categories: clear, smash, and drop. Preliminary experiments showed that three-way classification within the early anticipation window is extremely challenging due to similar preparatory kinematics among strokes, with all methods performing near chance level. Therefore, we collapse the three classes into a binary task consisting of aggressive strokes (clear and smash) versus soft shots (drop). This grouping is motivated by the defender’s primary tactical decision of whether to retreat for a deep shot or move forward for a net shot. The original class counts for the test set are 658 clear, 462 smash, and 444 drop clips, corresponding to 1120 aggressive and 444 soft clips after binary grouping. This yields a test set distribution of 42.1% clear, 29.5% smash, and 28.4% drop, or equivalently 71.6% aggressive versus 28.4% soft shots.

Direct joint optimization of these four terms from the start often leads to gradient conflicts, especially because early observation windows contain sparse cues. To address this, we adopt a curriculum learning strategy that progressively controls sample difficulty and loss weights. The difficulty of a training sample is determined by its ground-truth normalized trigger time $T_{norm}=T/T_{\max}$ (where $T_{\max}$ is the total duration of the rally) and the observation density (ratio of successfully extracted skeleton frames to the expected number of frames in that interval). These difficulty labels are used only during training. Training samples are divided into three difficulty levels based on these two factors: easy (short trigger time, high density), medium, and hard (long trigger time, low density). A smaller normalized trigger time and higher density correspond to easier difficulty.

Training proceeds in three stages. In the first stage, which occupies the initial 30% of total training epochs, only easy samples are used and the loss includes only the trajectory and classification terms, omitting $L_{time}$ and $L_{reg}$. This forces the model to learn stable temporal patterns from the most reliable cues. In the second stage, covering the next 30% of epochs, medium and hard samples are gradually mixed in and the weights of $L_{time}$ and $L_{reg}$ are linearly increased from zero to their final values. In the final stage, the remaining 40% of epochs are trained on the full dataset, with all loss terms at full weight. This progressive scheme prevents early domination by difficult samples or noisy gradient directions, leading to more stable convergence and better final performance, as validated in our ablation experiments.

### 3.6. Algorithmic Summary and Computational Complexity

To clarify the data flow and calling sequence of the proposed framework, we summarize the inference procedure of CT-ACI in Algorithm 1. The algorithm takes as input the full observation sequence $O={\left\{\left(\tau_{i},o_{i}\right)\right\}}_{i=1}^{N}$, the defender state sampled at observation times ${\left\{r\left(\tau_{i}\right)\right\}}_{\tau_{i}\leq t_{\mathrm{obs}}}$, and a cutoff time $t_{\mathrm{obs}}$. It returns the predicted trigger time $\hat{T}$, the intention category $\hat{y}$, and the predicted defender trajectory $\hat{r}\left(t\right)$ for $t>\hat{T}$.
**Algorithm 1** CT-ACI Inference**Input:** Observation sequence $O={\left\{\left(\tau_{i},o_{i}\right)\right\}}_{i=1}^{N}$; defender kinematic state ${\left\{r\left(\tau_{i}\right)\right\}}_{\tau_{i}\leq t_{\mathrm{obs}}}$; cutoff time $t_{\mathrm{obs}}$. **Output:** Predicted trigger time $\hat{T}$; intention $\hat{y}$; future trajectory $\hat{r}\left(t\right)\;\left(t>\hat{T}\right)$.
  1:$z\left(0\right)\leftarrow \phi_{\mathrm{enc}}\left({\left\{o_{i}\right\}}_{i=1}^{k}\right)$       ▹ Initialize state via temporal encoder  2:**for** i=1 to *N* such that $\tau_{i}\leq t_{\mathrm{obs}}$ **do**  3:      $z\left(\tau_{i}^{-}\right)\leftarrow \mathrm{ODESolve}\left(f_{\phi },z\left(\tau_{i-1}^{+}\right),\left[\tau_{i-1},\tau_{i}\right]\right)$       ▹ Continuous evolution  4:      $h_{i}\leftarrow \mathrm{CausalAttn}\left({\left\{z\left(\tau_{j}^{-}\right)\right\}}_{j=1}^{i},o_{i}\right)$       ▹ Posterior via temporal-causal attention  5:      $z\left(\tau_{i}^{+}\right)\leftarrow z\left(\tau_{i}^{-}\right)+h_{i}$       ▹ State posterior update  6:      $\Psi \left(\tau_{i}\right)\leftarrow \int_{0}^{\tau_{i}}{∥z\left(s\right)∥}_{2}ds$       ▹ Accumulated evidence  7:      $\theta \left(\tau_{i}\right)\leftarrow \sigma \left(W_{\theta }r\left(\tau_{i}\right)+b_{\theta }\right)\cdot \left(\theta_{\max}-\theta_{\min}\right)+\theta_{\min}$       ▹ Adaptive threshold  8:      $p_{\mathrm{trig}}\left(\tau_{i}\right)\leftarrow \sigma \left(\beta \left(\Psi \left(\tau_{i}\right)-\theta \left(\tau_{i}\right)\right)\right)$       ▹ Trigger probability  9:      **if** $p_{\mathrm{trig}}\left(\tau_{i}\right)>0.5$ **then**10:            $\hat{T}\leftarrow \tau_{i}$       ▹ Predicted trigger time11:            $\hat{y}\leftarrow \mathrm{Classifier}\left(z\left(\tau_{i}^{+}\right)\right)$       ▹ Intention category12:            $\hat{r}\left(t\right)\leftarrow \mathrm{TrajectoryDecoder}\left(z\left(\tau_{i}^{+}\right)\right)$ for $t>\hat{T}$       ▹ Future trajectory13:            **return** $\hat{T},\hat{y},\hat{r}\left(t\right)$14:      **end if**15:**end for**16:**return** Classifier$\left(z\left(t_{\mathrm{obs}}\right)\right)$, TrajectoryDecoder$\left(z\left(t_{\mathrm{obs}}\right)\right)$       ▹ Fallback at cutoff time


The forward pass of CT-ACI involves two dominant costs. The neural ODE solver (Dopri5) adaptively integrates the dynamics $f_{\phi }$ over inter-observation segments. Let K be the total number of integration steps, which is roughly proportional to the number of observation nodes N and far smaller than the number of raw video frames. Evaluating $f_{\phi }$ (a multilayer perceptron (MLP) with two layers and hidden dimension comparable to $D_{z}$) costs $O(D_{z}^{2})$ per step, yielding an ODE complexity of $O(KD_{z}^{2})$. Memory overhead is constant in K due to the adjoint sensitivity method. The causal attention mechanism computes pairwise scores between each observation and its predecessors, requiring $O(N^{2}D_{z})$ time and $O(N^{2})$ memory for the attention matrix. The remaining components (adaptive threshold, trajectory decoder, regularization integrals) contribute at most $O(ND_{z})$ or $O(KD_{z})$ and are negligible; thus, the overall time complexity is $O(KD_{z}^{2}+N^{2}D_{z})$. In typical badminton rallies, both K and N are of order $10^{2}$, which is substantially smaller than the total video length, making the framework computationally practical while maintaining strong robustness to irregular sampling.

## 4. Results

### 4.1. Implementation Details

The neural ODE uses the Dopri5 adaptive-step solver with absolute and relative tolerances of $10^{-5}$. The dynamics network $f_{\phi }$ consists of two fully connected layers (128 hidden units, tanh activation). Causal attention employs four heads with per-head dimension $d=D_{z}/4$. The temporal encoder $\phi_{enc}$ uses a causal 1D convolution (kernel size 3) on the first k=5 observed frames. Training uses AdamW (initial learning rate $10^{-3}$, weight decay $10^{-4}$) with cosine annealing, and batch size 32 for 100 epochs. Curriculum learning follows the three-stage schedule in Section 3.5. The key hyperparameters are β=10.0 for the sigmoid trigger sharpness, $\theta_{\min}=0.1$ and $\theta_{\max}=2.0$ for the adaptive threshold bounds, and $\lambda_{1}=0.5$ and $\lambda_{2}=0.1$ for the time prediction and regularization loss weights, respectively. To construct the defender’s hip-center velocity $v_{\mathrm{smooth}}$, the raw velocity is first computed from consecutive available hip-center observations as $v_{\mathrm{raw}}\left(\tau_{i}\right)={∥p_{\mathrm{hip}}\left(\tau_{i}\right)-p_{\mathrm{hip}}\left(\tau_{i-1}\right)∥}_{2}/\left(\tau_{i}-\tau_{i-1}\right)$, where $p_{\mathrm{hip}}$ denotes the hip-center position in normalized image coordinates. A causal five-frame trailing moving average is then applied over the sequence of available observations, yielding $v_{\mathrm{smooth}}\left(\tau_{i}\right)=\frac{1}{|W_{i}|}\sum_{k\in W_{i}}v_{\mathrm{raw}}\left(\tau_{k}\right)$, where $W_{i}=\left\{i-4,\ldots ,i\right\}$ and $|W_{i}|$ is the number of available observations in this window. This corresponds to a smoothing window of approximately 167 ms at 30 FPS.

To characterize real-time deployability, we profiled CT-ACI on a single NVIDIA RTX 4090 GPU with batch size 1, using evaluation mode, ten warm-up passes, and fifty timed repetitions with CUDA synchronization. CT-ACI achieved a single-forward-pass latency of 18.14 ms, contained 4,205,783 trainable parameters, and required 60.53 MB of peak GPU memory during a 104-frame forward pass. These workstation-based measurements do not establish embedded deployability; further optimization through fixed-step solvers or distillation remains as future work.

### 4.2. Experimental Setup

All experiments are conducted on the VideoBadminton dataset, which consists of 497 complete match rallies. The dataset split was performed before sliding window sampling. The 497 rallies were first divided into training, validation, and test subsets using a 6:2:2 rally-level split. Sliding window sampling was then applied independently within each subset, so all overlapping clips from the same rally remained in the same subset and could not appear in different subsets; this protocol prevents clip-level leakage. After sampling, the dataset contained 7823 valid action clips, including 4694 training clips, 1565 validation clips, and 1564 test clips.

The 497 rallies originate from six singles matches. Each match contains two player slots, giving 12 unique match–player slots in total. Because the dataset does not provide player-level identities, the number of unique real athletes is unknown; therefore, we report match–player slots rather than unique player identities.

The training subset contains four matches and eight match–player slots. The validation subset contains two matches and four match–player slots. The test subset contains three matches and six match–player slots. The subset-level match–player slot counts sum to 18, which exceeds the unique total of 12 because three matches appear in more than one subset. One match contributes to both training and validation, two matches contribute to both training and testing, and no match contributes to both validation and testing. After removing these overlaps, the six source matches correspond to 12 unique match–player slots. At least four of the six test match–player slots, or 66.7%, are associated with matches also used in training. At least two of the four validation match–player slots, or 50.0%, are associated with matches also used in training.

This overlap has two implications. First, there is no rally-level or clip-level leakage, as overlapping clips from the same rally are never split across subsets. Second, because two matches appear in both training and test, while player identities are unavailable, the same real athlete may appear in both training and test clips. Therefore, the present evaluation characterizes generalization to unseen rallies and clips but not to unseen matches or players. A match-disjoint or player-disjoint evaluation is not used as the primary protocol, as only six source matches are available and player identifiers are absent. A post hoc match-level sensitivity analysis is reported in Section 4.5 to quantify the influence of match overlap without retraining.

We assess model performance along four complementary dimensions, reporting medians and interquartile ranges throughout to mitigate the influence of long-tailed distributions. For all comparative evaluations, we report the median across three independent runs and use the interquartile range or standard deviation as the dispersion measure, depending on the table. This choice is motivated by three considerations. First, the trigger-time error distribution is heavily right-skewed (mean 251.3 ms vs. median 151.6 ms), making the median a more robust central tendency estimator than the mean. Second, all methods are evaluated on the identical set of 1,564 test clips, so pairwise comparisons are based on aligned samples; a median difference exceeding the pooled IQR width provides a practically meaningful separation criterion that is insensitive to distributional assumptions. Third, each reported value is the median across three independent runs with different random seeds (42, 123, and 456). For the standard test condition, the seed-to-seed standard deviation for CT-ACI is below 5 ms for TTE and below 0.6% for accuracy, with comparable magnitudes for all other methods. These variations are substantially smaller than the performance gaps between CT-ACI and discrete baselines under severe missing frame conditions, indicating that initialization randomness does not dominate the comparisons.

To assess whether the observed performance gaps are statistically meaningful, we performed paired Wilcoxon signed-rank tests on the per-sample errors. This technique was chosen because all methods were evaluated on the identical 1564 test clips. All paired tests were two-sided and performed at the 0.05 significance level. Effect sizes are reported as rank-biserial correlations. Bootstrap tests used 10,000 resamples.

For the Layer 3 TTE comparison between CT-ACI and LSTM, the test yields p<0.001 with a rank-biserial correlation of r=0.35. For the overall accuracy comparison between CT-ACI and LSTM, the test yields p=0.012 with r=0.08. Bootstrap paired tests on the median difference yield a 95% confidence interval of [45.2,114.3] ms for the Layer 3 TTE advantage and [0.6,4.6] percentage points for the overall accuracy difference. Additional paired tests against the remaining baselines are reported in Section 4.4 and Section 4.9.

For the Layer 3 stratum, CT-ACI achieves a median TTE of 155.3 ms (IQR: 104.5–346.0 ms) versus 235.0 ms (IQR: 92.0–462.0 ms) for LSTM, with a seed-to-seed standard deviation of 9.2 ms, an order of magnitude smaller than the median difference of 79.7 ms. Together with the paired statistical tests, this distributional separation supports the practical meaningfulness of the long-horizon advantage on this layer. Therefore, we report both descriptive measures and paired statistical tests, with practical significance assessed using median differences and IQR overlap.

Trigger time accuracy is measured by the time-to-event error (TTE), defined for a test set of *M* samples with reference label $T_{i}$ and prediction ${\hat{T}}_{i}$ as follows:

(20) $$\mathrm{TTE}={\mathrm{median}}_{i}|{\hat{T}}_{i}-T_{i}|$$

reported in milliseconds. Trajectory prediction quality is evaluated by the average displacement error (ADE) and the final displacement error (FDE). Starting from the trigger time, positions are sampled every 10 ms over a fixed horizon of *L* points, with predicted trajectory $\hat{r}\left(t_{k}\right)$ and ground truth $r(t_{k})$:

(21) $$\mathrm{ADE}=\frac{1}{L}\sum_{k=1}^{L}{∥\hat{r}\left(t_{k}\right)-r\left(t_{k}\right)∥}_{2}$$

(22) $$\mathrm{FDE}=\|\hat{r}\left(t_{k}\right)-r\left(t_{k}\right){\|}_{2},$$

both reported in normalized image coordinate units (scaled by 100 for numerical convenience) as the median over the test set. As noted in Section 3.1, these values do not correspond to physical centimeters, and are meaningful only for relative comparisons across methods. The same caveat applies to the velocity thresholds used to define $T^{*}$, which are expressed in dimensionless normalized velocity units (NVU) rather than physical speeds. Finally, intention classification accuracy is computed as

(23) $$\mathrm{Accuracy}=\frac{1}{M}\sum^{M}1\left({\hat{y}}_{i}=y_{i}\right),$$

where $y_{i}$ and ${\hat{y}}_{i}$ denote the ground-truth and predicted binary intention categories: aggressive (clear/smash) versus soft (drop). The mapping from the original three-class labels (clear, smash, drop) to this binary task, along with class counts and underlying rationale, is described in Section 3.5.

The proposed CT-ACI model is compared against LSTM, GRU, Standard ODE, Causal GCN, and an adapted SkelVIT-style baseline. AntGPT [9] is excluded due to its RGB video-based LLM architecture and the unavailability of its sports-specific pre-trained weights, which preclude a fair comparison under our skeleton input setting. The original SkelVIT [8] is designed for action classification and does not output future trajectories. To enable fair comparison on our trigger-time regression and trajectory prediction tasks, we adapted its architecture by retaining the skeleton encoder while adding a trajectory prediction head to output future defender coordinates. This adaptation preserves SkelVIT’s core design principles while matching our multi-task output interface. All models follow the same training configuration detailed in Section 4.1 and are evaluated on identical data splits. For each method, three independent runs are performed with random seeds 42, 123, and 456; all reported values are medians across these runs. All models share identical pose inputs and T* labels from the same MediaPipe pipeline, so any pose estimation uncertainty affects all methods equally and does not bias the comparative conclusions. Experiments are implemented in PyTorch 2.0 and executed on an NVIDIA RTX 4090 graphics processing unit (GPU).

### 4.3. Robustness Under Realistic Sensing Constraints

We evaluate model robustness under two realistic degradation scenarios. First, skeleton frames are randomly dropped at rates from 0% to 90% during inference. Figure 2 reports the resulting TTE under increasing missing frame ratios. CT-ACI maintains stable TTE up to 50% missing (151.3 ms) and degrades only 30.9% at 90% missing (from 151.6 ms to 198.5 ms). Standard ODE, which shares the neural ODE backbone but lacks the temporal–causal attention mechanism, degrades 46.8% (from 168.3 ms to 247.1 ms), substantially less than LSTM (344.0%, from 60.4 ms to 268.2 ms) and GRU (426.2%, from 61.8 ms to 325.2 ms).

Together, these results suggest two complementary sources of robustness. First, neural ODE dynamics reduce degradation from the 344%–426% range to 46.8%, indicating that continuous-time integration over gaps provides inherent robustness. Second, temporal–causal attention further reduces degradation from 46.8% to 30.9%, suggesting effective incorporation of sparse observations. Thus, continuity over gaps and attentive updating jointly appear to contribute to the observed robustness. LSTM and GRU exhibit sharp degradation beyond 30% missing because they rely on fixed-step temporal representations.

To further separate the sources of this robustness, we compare CT-ACI with Standard ODE and discrete recurrent baselines at the 90% missing frame level.

As shown in Table 1, Standard ODE substantially outperforms LSTM and GRU at 90% missing (46.8% vs. 344.0% and 426.2% TTE increases), indicating that continuous dynamics provide substantial robustness. CT-ACI further improves upon Standard ODE (30.9% vs. 46.8%), highlighting the attention mechanism’s additional contribution. Both components contribute jointly: the ODE provides continuity over gaps, while the attention mechanism supports effective incorporation of sparse observations. This disentanglement suggests that the robustness of CT-ACI is likely attributable to the synergy between continuous-time integration and temporally constrained attention rather than to either component in isolation.

Second, we randomly permute the timestamps of input sequences, then compare per-sample TTE before and after shuffling (Figure 3). CT-ACI degrades gracefully, with relative TTE increase remaining under 50%, while LSTM and GRU both exceed 200% degradation, suffering catastrophic failures on a substantial fraction of samples. The continuous dynamics propagate the intention state forward in a physically consistent manner regardless of observation order; only the posterior updates at observation times are affected by the permutation.

The timestamp permutation experiment is intended as a controlled synthetic fault injection test; in real competitions, observation sequences are strictly chronological, so timestamp corruption caused by packet reordering or poorly synchronized cameras is uncommon and typically affects only a small fraction of frames. The experiment is intended to quantify the model’s reliance on temporal ordering and assess whether continuous-time dynamics can partially compensate for order violations through integration of the learned vector field. Therefore, the degradation observed under full permutation represents an upper bound on the sensitivity to temporal metadata errors; under this diagnostic interpretation, the experiment supports the claim that CT-ACI is less sensitive to ordering disruptions than discrete recurrent models, without necessarily implying that chronological order is dispensable in deployment.

These results indicate that CT-ACI retains predictive capability under conditions that render discrete baselines inoperable. The robustness appears to stem from the physical continuity constraint in the neural ODE backbone, which is not readily achieved by simply increasing the capacity of discrete architectures or tuning their hyperparameters.

### 4.4. Performance Stratified by Prediction Difficulty

To examine the effect of prediction horizon, we stratify test samples into four layers using the normalized reference trigger time: Layer 1 (0–0.1), Layer 2 (0.1–0.3), Layer 3 (0.3–0.7), and Layer 4 (0.7–1.0). Most samples fall into Layers 1–2, reflecting the short trigger times typical of competitive badminton. Figure 4 compares CT-ACI and LSTM across these strata. Discrete models hold a clear advantage on short horizons (Layers 1–2), where local temporal cues suffice and error accumulation is negligible. A critical reversal emerges on Layer 3, where CT-ACI achieves a median TTE of 155.3 ms (IQR: 104.5–346.0 ms) while LSTM achieves 235.0 ms (IQR: 92.0–462.0 ms), corresponding to a 33.9% median reduction. This 33.9% median reduction is statistically significant under a paired Wilcoxon signed-rank test (p<0.001, rank-biserial r=0.35) and a bootstrap paired test (95% CI for median TTE difference: [45.2,114.3] ms). More importantly, the upper quartile for CT-ACI (346.0 ms) falls below the third quartile for LSTM (462.0 ms), while their IQR ranges exhibit only limited overlap. This distributional separation, together with the paired statistical tests reported in Section 4.2 and the minimal seed-to-seed standard deviation on this layer (9.2 ms), supports the practical meaningfulness of the long-horizon advantage of CT-ACI. For completeness, paired Wilcoxon signed-rank tests between CT-ACI and the remaining baselines on Layer 3 TTE yield rank-biserial correlations of r=0.31 (GRU), r=0.29 (Standard ODE), r=0.26 (Causal GCN), and r=0.33 (SkelVIT), all with p<0.01. CT-ACI maintains a lower TTE on Layer 4 as well, although sample sizes there are limited.

These results reveal a complementary relationship between the two paradigms: discrete recurrent architectures are preferable for short-horizon, high-confidence anticipation, where local temporal cues are sufficient; in contrast, CT-ACI becomes more competitive as the prediction horizon increases, showing lower median TTE in Layers 3–4. These findings delineate the practical scope of CT-ACI. While it is not intended to replace discrete models at all timescales, it can improve stability when anticipation horizons are extended or temporal uncertainty is high.

To complement the TTE-based stratification, Table 2 reports ADE and FDE across difficulty layers.

The layer-wise results show that LSTM achieves lower ADE/FDE on the easy and medium layers, consistent with its short-term modeling strength. However, CT-ACI achieves lower ADE and FDE on the more difficult layers L3 and L4, with the gap widening in L4, where ADE is 7.6 versus 9.0 and FDE is 5.4 versus 8.0. LSTM’s ADE increases from 3.7 to 9.0 (+5.3), whereas CT-ACI’s ADE increases from 5.1 to 7.6 (+2.5), indicating that CT-ACI maintains better long-horizon trajectory stability.

### 4.5. Match-Level Sensitivity Analysis

To quantify the influence of the match overlap discussed in Section 4.2, we perform a post hoc match-level sensitivity analysis on the test set. Because player identifiers are unavailable and only six source matches exist, this analysis is not a substitute for player-level cross-validation; rather, it tests whether the main conclusions are driven by the two test matches that also appear in training.

The test set contains three source matches; two overlap with the training set and one appears only in the test set. We partitioned the 1,564 test clips according to their source match into a test-overlap subset and a test-holdout subset. No retraining was performed. Predictions from the same CT-ACI and LSTM models were grouped by match, and TTE and accuracy were recomputed for each subset. Table 3 reports the per-method sensitivity to the holdout match.

Both methods degrade on the test-holdout subset relative to the test-overlap subset. CT-ACI shows a smaller relative TTE increase than LSTM (9.5% versus 12.1%) and a smaller relative accuracy decrease (2.1% versus 2.6%). The gaps between the methods remain stable, with LSTM retaining a lower TTE in both subsets and CT-ACI retaining a higher accuracy in both. This indicates that the main conclusions are not driven solely by the shared matches; because the test-holdout subset comes from only one source match, its estimate has high variance, and should be interpreted as a qualitative sensitivity check rather than a precise generalization estimate. A full leave-one-match-out cross-validation was not used as the primary protocol, since with six matches each fold would remove a large fraction of the data, while the absence of player identifiers precludes player-level stratification.

### 4.6. Ablation Study

We evaluate five model variants on the test set to quantify component sensitivity across timing and classification metrics. The results are summarized in Figure 5 and Table 4. Removing the temporal–causal attention mask produces the largest increase in TTE, from 151.6 ms to 170.8 ms, indicating that temporal–causal masking mainly supports trigger-time precision. In contrast, replacing the continuous-time encoder with a GRU causes the largest decrease in Accuracy, from 78.7% to 74.1%, while removing curriculum learning also reduces accuracy to 75.5%. These results indicate task-specific component sensitivity. The temporal–causal mask contributes most strongly to temporal precision, whereas the neural ODE encoder and curriculum strategy contribute more substantially to intention classification. Therefore, the full model benefits from the combination of continuous dynamics, temporally constrained attention, adaptive triggering, and staged optimization rather than from a single dominant component.

Numerical ablation results are summarized in Table 4. TTE values are reported as median with interquartile range (Q1, Q3). Accuracy values are reported as mean with 95% confidence interval (lower, upper). Lower TTE and higher accuracy indicate better performance.

### 4.7. Sensitivity Analysis of the Trigger-Time Threshold

To assess whether the reported TTE is sensitive to the primary velocity threshold, we varied $\theta_{\mathrm{primary}}$ from 0.10 to 0.50 NVU in steps of 0.05 NVU on the test set. For each θ, we regenerated the pseudo ground-truth labels $T^{*}\left(\theta \right)$ and computed the TTE between our fixed model predictions (trained with θ=0.3) and these alternative labels. Table 5 reports the median TTE and interquartile ranges. The results reveal a stable plateau between 0.25 and 0.35 NVU, where the median TTE varies by less than 11 ms (from 162.5 ms to 151.6 ms). Even at θ=0.35, the TTE (155.8 ms) deviates by only +2.8% from the trained threshold. Conversely, extreme thresholds (0.1 or 0.5 NVU) introduce systematic bias due to physically implausible early or late trigger definitions. This analysis confirms that the model’s performance is not an artifact of a specific threshold choice.

### 4.8. Pose Estimation Selection and Validation

#### 4.8.1. Hip Landmark Confidence Statistics

For the hip landmarks, which are directly used to compute defender velocity and $T^{*}$, we report joint-specific confidence statistics to assess input reliability. For each parsed frame, MediaPipe outputs a confidence score for every detected landmark. The hip-center position is computed as the midpoint between the two hip landmarks, and the hip confidence is defined as the minimum of the confidence scores of these two landmarks. The failure threshold is set to 0.5, consistent with the low-confidence criterion used for handling missing frames throughout the pipeline. Table 6 reports the resulting statistics across all parsed frames from the training, validation, and test splits.

The hip-center mean confidence is 0.87 with a standard deviation of 0.09, and the failure rate is 2.3%. The median is 0.91, indicating that the confidence distribution is skewed toward high values. The 5th percentile is 0.62, which remains above the failure threshold of 0.5, confirming that the hip estimates are reliable for the vast majority of frames even in the lower tail. These statistics indicate that the hip landmarks are detected reliably across the dataset.

Differentiating landmark positions across frames propagates pose estimation noise into velocity, $T^{*}$, ADE, and FDE. Three factors mitigate this concern. First, a five-frame trailing moving average (∼167 ms at 30 FPS) attenuates high-frequency jitter. Quantitatively, this smoothing reduces the frame-to-frame standard deviation of hip-center velocity from 0.084 to 0.031 NVU, corresponding to a 63% reduction in high-frequency jitter. Second, all compared methods share identical pose inputs and $T^{*}$ labels, ensuring symmetric bias across models. Third, frames with hip confidence below 0.5 are treated as missing observations and integrated over by the neural ODE solver. Any residual systematic bias from the 2D detector, such as a consistent temporal lag under fast motion, is shared across all compared methods and so does not affect the relative comparison.

#### 4.8.2. Unified Accuracy Evaluation

To compare the accuracy of the six pose estimation frameworks under a single evaluation protocol, we conducted a unified accuracy evaluation on a stratified subset of 100 test clips, with the number of clips drawn from each difficulty layer proportional to the layer size. ViTPose-B was used as a practical reference because it achieves the highest reported COCO AP among the compared frameworks, but does not represent ground truth. For each framework, we computed the hip-center position as the midpoint between the two hip landmarks and calculated the mean normalized Euclidean distance to the reference hip-center position. All frameworks were run with identical input resolution and preprocessing. Table 7 reports the resulting hip-center errors together with CPU inference speed and deployment characteristics. Lower error indicates higher accuracy.

The results show that ViTPose-B achieves the lowest error by construction, consistent with its highest reported COCO AP. RTMPose-m achieves the second-lowest error among all frameworks and the lowest error among CPU-friendly methods. AlphaPose (0.6.0) and OpenPose (v1.7.0) show moderate errors, while MediaPipe and MoveNet (Thunder variant, TF Hub version 3) exhibit slightly larger errors. Despite this, MediaPipe remains competitive given its much lower computational cost and faster CPU inference. This unified evaluation confirms that the accuracy–speed trade-off observed in the reported literature also holds under a single evaluation protocol on the present dataset.

#### 4.8.3. Cross-Estimator Consistency of Trigger-Time Labels

To assess whether the operational trigger-time labels $T^{*}$ are sensitive to the choice of pose estimation framework, we conduct a cross-framework consistency analysis on a stratified random subset of 100 test clips. The number of clips drawn from each difficulty layer is proportional to the layer size, as defined in Section 4.4. The five alternative pose estimators in the evaluation are ViTPose, RTMPose, AlphaPose, OpenPose, and MoveNet. Among these, RTMPose-m [35] is selected as the primary alternative because it combines competitive accuracy (75.8% AP on COCO) with CPU-friendly inference speed (90+ FPS), has been validated against IMU reference systems for hip kinematics with an error of 3.1° ± 1.8° [36], and shares the single-person pose estimation paradigm with MediaPipe.

For each estimator, we re-ran the full preprocessing pipeline consisting of frame extraction, pose inference, hip-center computation, five-frame smoothing, and the identical thresholding rule (0.3 NVU primary, 0.1 NVU fallback, peak-speed fallback) to generate the corresponding $T^{*}$ labels. Table 8 reports the agreement between MediaPipe-derived and estimator-derived $T^{*}$ labels. Across all five estimators, the correlation remains consistently high, ranging from 0.956 (MoveNet, Layer 4) to 0.995 (ViTPose, Layer 1). The correlation is highest for ViTPose, which achieves the highest COCO AP among the compared estimators, and lowest for MoveNet, consistent with its lower absolute accuracy. Across all estimators, the correlation decreases slightly from Layer 1 to Layer 4, which is attributable to slower velocity evolution near the threshold in long-horizon samples. Importantly, all correlations remain above 0.95, indicating that the discrepancies between MediaPipe-derived and estimator-derived labels are not systematic, as the relative ordering of samples is preserved across all estimators and all difficulty layers. Since all models share identical $T^{*}$ labels derived from the same pipeline and since the label agreement across estimators is substantially stronger than the performance gaps between methods reported in the main results, the relative ranking of methods is not an artifact of the pose estimation pipeline.

### 4.9. Unified Performance Summary

Table 9 summarizes the performance of CT-ACI and all compared baselines on the test set under 0% and 90% missing frames. The 0% missing frame setting provides the standard test set reference, where LSTM and GRU achieve lower TTE owing to the availability of dense temporal observations. However, this advantage is substantially reduced when the observation sequence becomes severely incomplete. Under the 90% missing frame setting, CT-ACI achieves the lowest TTE and maintains the most balanced performance across binary intention classification and trajectory prediction metrics. This pattern indicates that the benefit of CT-ACI lies primarily in robust intention anticipation from sparse and irregular temporal evidence rather than in optimizing short-term timing precision under fully observed inputs. Table 9 reports all values with standard deviations across the three independent runs, providing explicit uncertainty estimates. The seed-to-seed variability is substantially smaller than the performance gaps between CT-ACI and discrete baselines under severe missing-frame conditions, confirming that the reported advantages are not dominated by initialization randomness.

Figure 6 projects each method onto the plane defined by TTE and binary intention classification accuracy. LSTM and GRU achieve the lowest TTE, reflecting their strength in short-term temporal modeling. CT-ACI attains the highest intention classification accuracy (78.7%), marginally above LSTM (76.1%) and GRU (75.9%). The 2.6 percentage-point accuracy advantage over LSTM is statistically significant under a paired Wilcoxon signed-rank test (p=0.012, r=0.08) and a bootstrap paired test (95% CI: [0.6,4.6] percentage points), although the effect size is small; therefore, this figure should be interpreted as a coarse early warning signal rather than as evidence of fine-grained classification superiority, consistent with the cautionary note below. CT-ACI lies on the Pareto front, meaning that no other method simultaneously achieves higher accuracy at a lower or equal TTE. In trajectory prediction, CT-ACI achieves ADE = 6.3 and FDE = 4.2 (in scaled image coordinate units), comparable to LSTM (5.1/3.4) and GRU (5.6/4.7) while surpassing Causal GCN (6.6/5.3), Standard ODE (6.8/5.6), and SkelVIT (7.9/6.6). This positions CT-ACI as a balanced multi-task framework with the highest classification accuracy among the compared methods and trajectory prediction on par with the strongest discrete baselines.

To quantify whether the accuracy advantage of CT-ACI over each baseline is statistically meaningful, we performed paired Wilcoxon signed-rank tests under the standard test condition. Table 10 reports the *p*-values and rank-biserial effect sizes. All comparisons are statistically significant at the 0.05 level, although the effect sizes vary. The largest effect is observed against Standard ODE (r=0.28) and the smallest against SkelVIT (r=0.09).

These results reflect a fundamental trade-off, with CT-ACI prioritizing robustness and long-term temporal validity over short-term precision, leading to a higher TTE in the 0% missing frame setting. This behavior is a structural consequence of its non-anticipative attention mask and continuous-time dynamics. No single method dominates all dimensions, indicating that the choice of architecture should depend on whether the application prioritizes short-term timing precision or resilience under real-world sensing uncertainty.

The reported 78.7% binary accuracy (aggressive (clear/smash) vs. soft (drop)) is only marginally higher than the LSTM baseline, and should be interpreted cautiously. Its practical relevance depends on the temporal context in which the prediction is used. In real-time badminton, the binary distinction between aggressive strokes and soft drops is a critical perceptual decision that determines whether the defender should retreat for a deep shot or move forward for a net shot. When combined with an average lead time exceeding 150 ms, this level of accuracy can provide an early candidate signal for elite training and post-match analysis, even though errors remain in approximately one-fifth of cases. This result should not be interpreted as evidence of fine-grained stroke recognition or autonomous tactical competence; instead, its value lies in providing a coarse early warning signal under a physical causal mask, whereas standard offline models may benefit from implicit future context and overestimate their online deployability.

Accordingly, the current results support CT-ACI as a temporally non-anticipative and degradation-robust anticipation framework that can generate early candidate intentions; they do not establish tactical classification or generally superior recognition capability.

### 4.10. Qualitative Analysis

Figure 7 reports the distribution of absolute trigger-time prediction errors on the test set. Reference trigger times range from 0.033 s to 1.567 s, with a median of 0.300 s. The CT-ACI error distribution exhibits a pronounced right tail, with a median of 151.6 ms, mean of 251.3 ms, standard deviation of 264.4 ms, and maximum of 1195.5 ms. For comparison, LSTM achieves a median of 60.4 ms, mean of 205.3 ms, standard deviation of 266.8 ms, and maximum of 1291.5 ms.

The complete distribution statistics are summarized in Table 11. These statistics confirm that CT-ACI has a longer right tail, with a mean approximately 100 ms higher than its median, while the standard deviations are comparable between the two methods (264.4 ms vs. 266.8 ms). Notably, the maximum error of CT-ACI (1195.5 ms) is slightly lower than that of LSTM (1291.5 ms). This trade-off is consistent with CT-ACI’s design priority, as its principal benefit lies in robustness under irregular or missing observations (Figure 2 and Figure 4) rather than in minimizing central-tendency errors under the original test condition.

A representative case featuring a long rally is shown in Figure 8. The true trigger time is 333.3 ms. CT-ACI predicts 330.5 ms, an error of only 2.8 ms, substantially outperforming LSTM at 10.6 ms and GRU at 269.0 ms. This case illustrates that continuous-time modeling can achieve high precision when observations are sufficiently dense and kinematic cues are unambiguous.

Figure 9 presents a long-horizon failure case with only two reliable pre-trigger observations and near-static preparation with sparse reliable cues. In panel (a), the red dots mark the two reliable pre-trigger observations (Obs. 1 and Obs. 2), and the dashed vertical line indicates the trigger point. In panel (b), the red dashed line indicates the 1000 ms failure threshold. All three methods exceed this threshold, demonstrating that weak early evidence can defeat both continuous-time and discrete models.

## 5. Discussion

### 5.1. Applicability Under Missing Defender Posture

A practical concern for deploying CT-ACI in real matches is the availability of the defender’s kinematic state r(t). While our framework assumes that r(t) is observable (e.g., from a multi-camera system or inertial sensors), single-view broadcast footage often fails to simultaneously capture both players with sufficient resolution.

To address this, we propose a simple but effective fallback mechanism. When r(t) is unavailable, the adaptive threshold θ(t) defined in Equation (13) degenerates to a fixed learnable scalar $\theta_{0}$:

(24) $$\theta \left(t\right)=\theta_{0},\;\mathrm{if}\;r\left(t\right)\;\mathrm{is}\;\mathrm{missing}$$

and the triggering condition reduces to $\Psi \left(t\right)\geq \theta_{0}$. In this degraded mode, the model loses the ability to adjust the decision threshold based on the defender’s preparation state, but critically, the core continuous-time dynamics (neural ODE) and temporal–causal attention mechanism remain fully operational. The event-driven decision is then driven purely by the accumulated causal evidence from the opponent’s motion, which still provides a functionally useful early warning signal.

Furthermore, when r(t) is partially available (e.g., intermittent occlusion), we recommend a simple zero-order hold imputation in which the last valid posture is propagated forward until new data arrive. This heuristic, while not theoretically optimal, ensures numerical stability of the integral trigger without requiring retraining of the entire model.

We emphasize that the selection between the full adaptive mode and the degraded fixed-threshold mode is a configurable hyperparameter at inference time, determined by the specific sensing setup of the deployment environment. This modular design ensures that CT-ACI remains applicable across a wide range of practical scenarios from laboratory-grade multi-view capture to single-camera broadcast analysis.

### 5.2. Limitations and Scope

Several limitations should be considered when interpreting the present results. First, the timestamp permutation experiment is a synthetic fault injection diagnostic, not an ecological simulation. Since actual match observations are chronological and timestamp corruption is uncommon, these results only represent evidence of sensitivity to ordering metadata errors.

Second, the Dopri5 adaptive ODE solver introduces computational overhead, requiring 16.92 ms and 20 function evaluations per forward pass at batch size 32 on an NVIDIA RTX 4090 GPU. This performance is sufficient for offline analysis, but does not establish embedded deployability; further optimization through fixed-step solvers, tolerance adjustment, or model distillation remains unvalidated.

Third, the preprocessing pipeline depends on MediaPipe-derived 2D pose estimates. Trigger-time labels and defender trajectories are computed using a fixed 0.3 NVU hip-center speed threshold, while ADE and FDE are reported in normalized image coordinate units (scaled by 100 for numerical convenience) rather than calibrated physical distances. Because VideoBadminton provides no camera intrinsics, court homography, or 3D reconstruction, the model inherits biases from the 2D landmark detector and pixel-scale approximation. While the confidence statistics and the cross-estimator consistency analysis reported in Section 4.8 are the most direct available proxies for pose reliability under the present dataset constraints, they do not constitute a full 3D pose accuracy evaluation such as MPJPE, which cannot be computed without 3D ground truth.

Fourth, the reported results characterize the VideoBadminton benchmark and do not establish player-level or population-level generalization. As detailed in Section 4.2, the 497 rallies originate from six singles matches, giving 12 unique match–player slots in total. Because player identifiers are not released, the number of unique real athletes is unknown and may be smaller than 12 if the same athlete appears in multiple matches. The per-match rally-level split prevents clip-level leakage, but three of the six matches contribute rallies to more than one subset. In particular, two matches contribute to both training and test, so at least four of the six test match–player slots, or 66.7 percent, are associated with matches also used in training. Our post hoc match-level sensitivity analysis in Section 4.5 shows that both methods degrade on the test-holdout subset, but the between-method gaps remain stable and the relative ranking is preserved. Even so, this analysis cannot establish generalization to unseen players, as player-level identities are unavailable and the test-holdout subset comes from only one match. Thus, the current results assess generalization to unseen rallies and clips, not to unseen matches or players. All experiments are confined to singles play. Doubles matches introduce additional players, occlusions, rotations, interceptions, and inter-player tactical interactions that are not covered by the current single-opponent, single-defender formulation.

Finally, while the architecture is sensor-agnostic in principle, its neural ODE dynamics, attention mechanism, and threshold hyperparameters were tuned specifically for badminton kinematics. Transfer to other racquet sports, live deployment, and integration with event cameras or wearable IMUs would require domain-specific retuning and revalidation.

### 5.3. Sensor Design Insights and Future Directions

From a sensor systems engineering perspective, CT-ACI offers several implications for visual sensing in sports analytics. The event-driven threshold trigger decouples prediction frequency from sensor frame rate, suggesting that visual sensing systems may operate in a lower-power mode and activate more frequently only when accumulated evidence approaches the decision threshold. The neural ODE backbone integrates over nonuniform inter-frame intervals without resampling or interpolation, thereby reducing dependence on strict hardware-level synchronization across cameras. In addition, the inference profile of 18.14 ms per forward pass, 4.2M parameters, and 60.5 MB peak GPU memory suggests feasibility on moderately capable edge devices for offline post-match analysis, although real-time on-court deployment would require further optimization.

Future work should examine four directions. Event cameras may provide timing cues at high temporal resolution even under motion blur, while wearable IMUs could supply occlusion-robust kinematic signals and improve intention classification under severe visual occlusion. Deployment on edge computing devices requires systematic validation of throughput, power consumption, and solver tolerance trade-offs under field conditions. Extending the method to doubles play and other racquet sports would further require domain-specific data collection, retuning of neural ODE dynamics, and revalidation of the adaptive threshold mechanism.

## 6. Conclusions

This paper has proposed the CT-ACI framework for intention-driven anticipation in badminton, combining neural ODEs, temporal–causal attention, and adaptive integral triggering. The proposed framework handles irregular sampling, prevents backward leakage, and enables event-driven predictions. On VideoBadminton, CT-ACI maintains stable performance under severe missing frames and shows lower long-horizon median TTE than LSTM on the more difficult Layers 3–4. Ablation confirms task-specific component sensitivity, with the temporal–causal mask most strongly supporting trigger-time precision and the neural ODE encoder and curriculum strategy primarily affecting classification accuracy. These results demonstrate that CT-ACI is best interpreted as a balanced multi-task framework providing early candidate signals for downstream analysis, with $T^{*}$ serving solely as an operational proxy for method comparison.

## Figures and Tables

**Figure 1:**
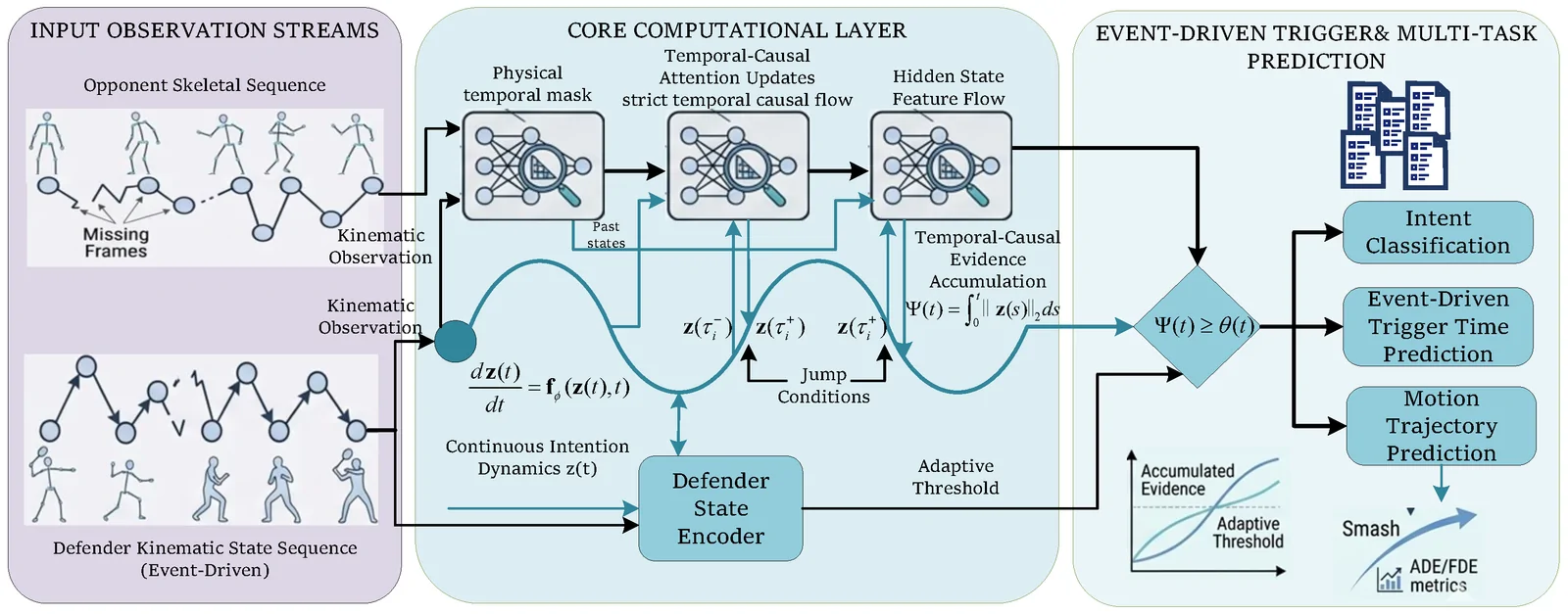
Overview of the proposed Continuous-Time Event-Driven Temporal–Causal Attention (CT-ACI) framework. The architecture processes non-uniform opponent skeleton sequences and defender states through a neural ordinary differential equation (neural ODE) and a physical temporal–causal attention mask. An adaptive integral mechanism then triggers event-driven multi-task predictions, including intention classification, trigger-time estimation, and future defensive trajectory prediction.

**Figure 2:**
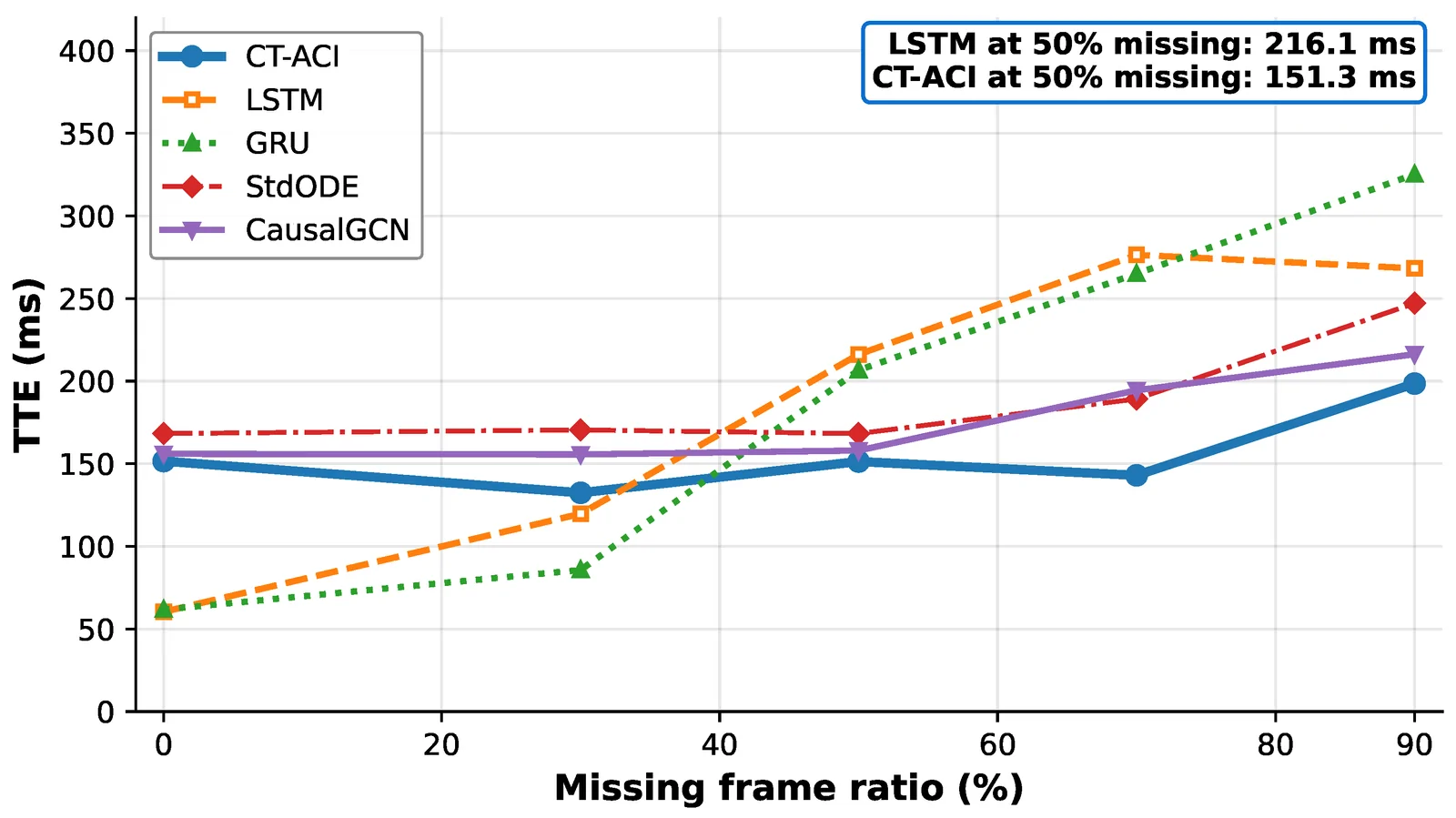
TTE of CT-ACI, Standard ODE, LSTM, and GRU under missing frame ratios from 0% to 90% during inference. CT-ACI maintains stable TTE up to 50% missing (151.3 ms) and degrades only 30.9% at 90% missing (151.6 ms to 198.5 ms). Standard ODE degrades 46.8% (168.3 ms to 247.1 ms), substantially less than LSTM (344.0%, 60.4 ms to 268.2 ms) and GRU (426.2%, 61.8 ms to 325.2 ms). This two-level comparison separates the contribution of the neural ODE dynamics (Standard ODE vs. LSTM/GRU) from the additional contribution of the temporal–causal attention mechanism (CT-ACI vs. Standard ODE).

**Figure 3:**
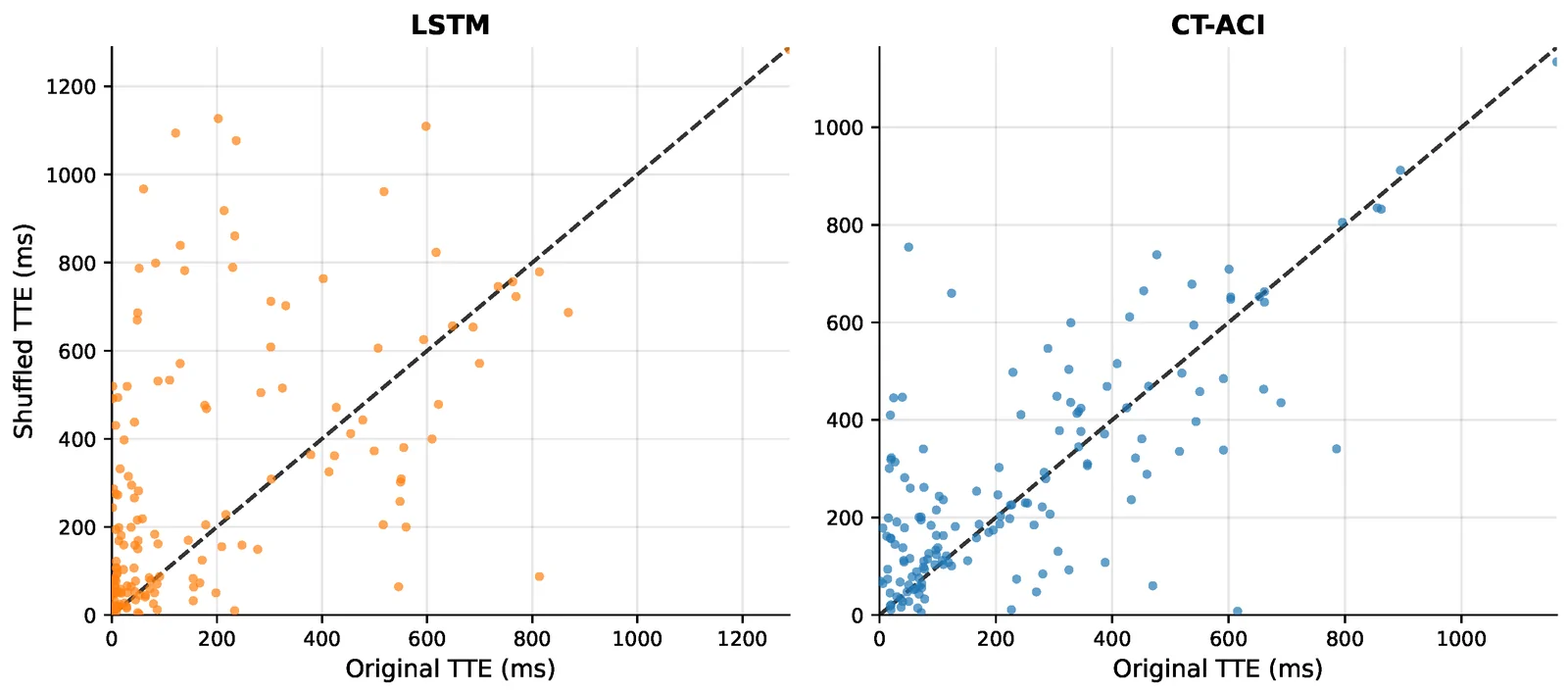
Per-sample TTE before and after temporal shuffling for LSTM and CT-ACI. This experiment provides a synthetic diagnostic test that diagnoses the sensitivity to temporal ordering. Each point is one test sample; the diagonal marks unchanged performance. For LSTM, the points scatter far above the diagonal, indicating catastrophic degradation on a large fraction of samples. For CT-ACI, the points remain tightly clustered around the diagonal, showing that the continuous dynamics preserve predictive structure even when the observation order is disrupted.

**Figure 4:**
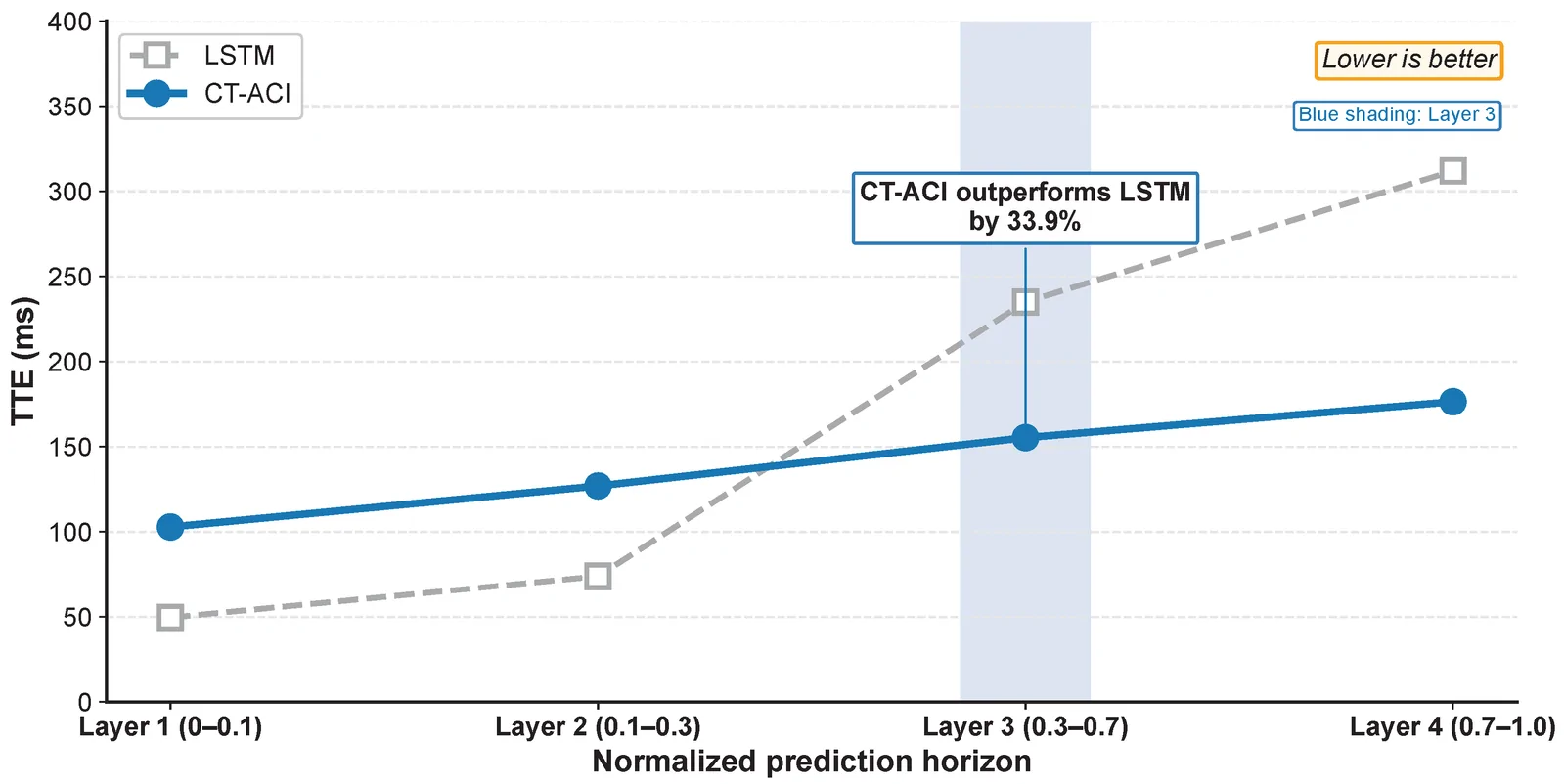
TTE of CT-ACI and LSTM across four difficulty layers defined by normalized trigger time. The horizontal axis denotes the four layers from Layer 1 (short horizon) to Layer 4 (long horizon), and the vertical axis denotes TTE in milliseconds, with lower values indicating better performance. The light-blue shaded region highlights Layer 3, where CT-ACI outperforms LSTM by 33.9%. The pale-yellow annotation indicates that lower TTE is better.

**Figure 5:**
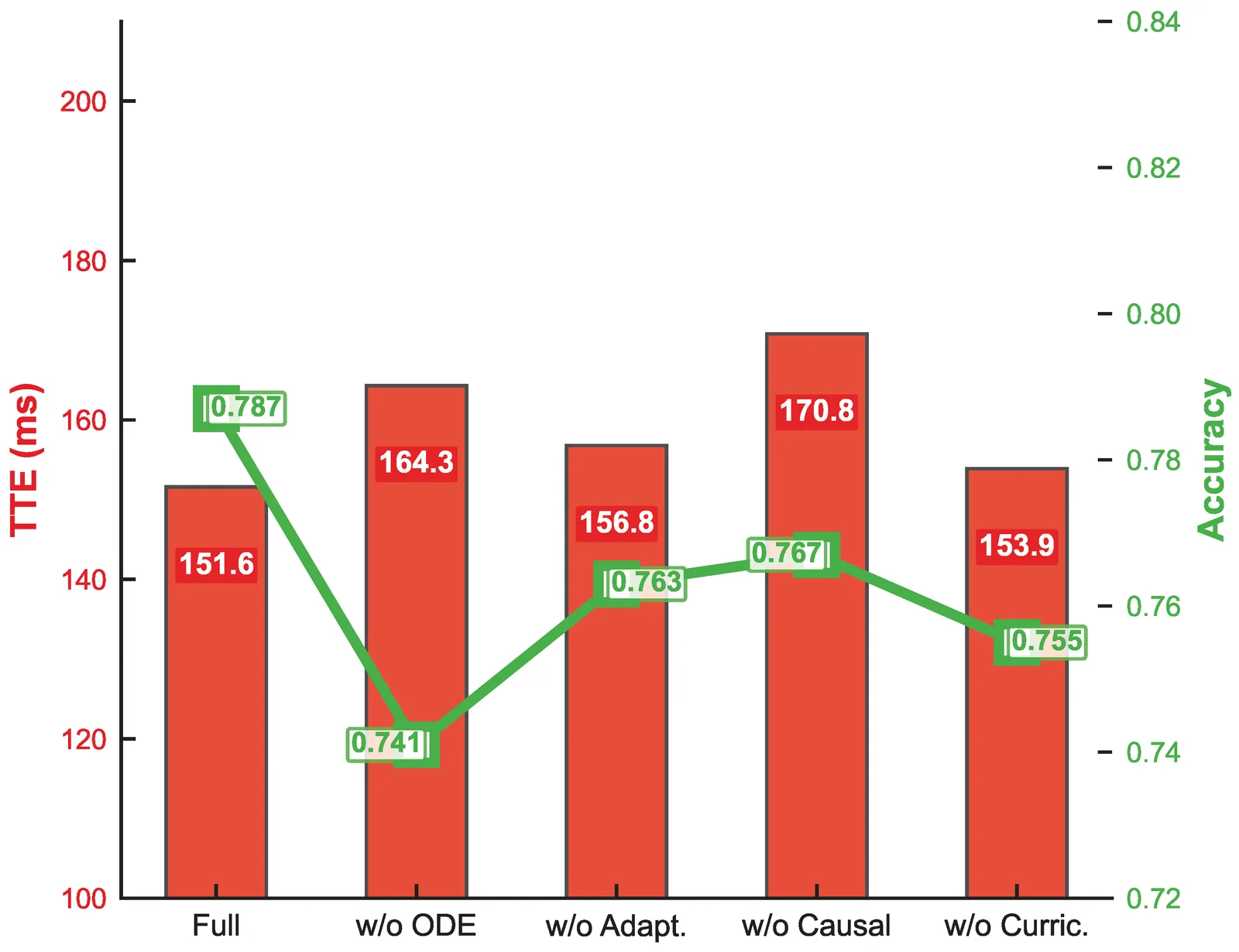
Component-wise ablation results on the test set. Each bar shows the median TTE (in milliseconds) for each ablation variant; lower values indicate better timing accuracy.

**Figure 6:**
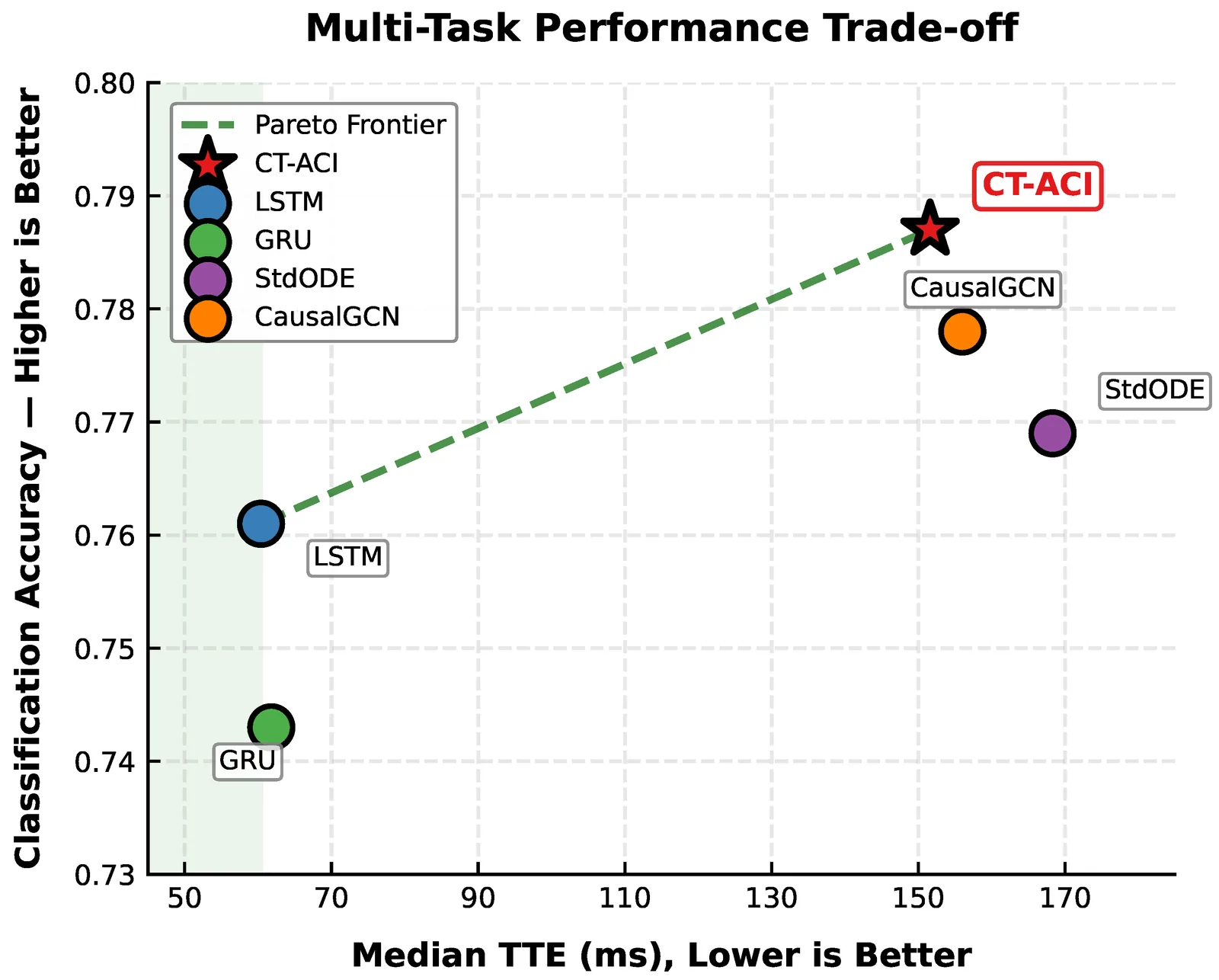
Pareto front in the TTE–accuracy plane on the test set. Each marker denotes one method. CT-ACI lies on the Pareto front with the highest accuracy of 78.7% and a TTE of 151.6 ms, indicating that no other method simultaneously achieves lower TTE and higher accuracy. LSTM and GRU occupy the region with low TTE and low accuracy, while Causal GCN occupies the high-TTE region. The shaded area below the Pareto front is dominated by Pareto-optimal methods.

**Figure 7:**
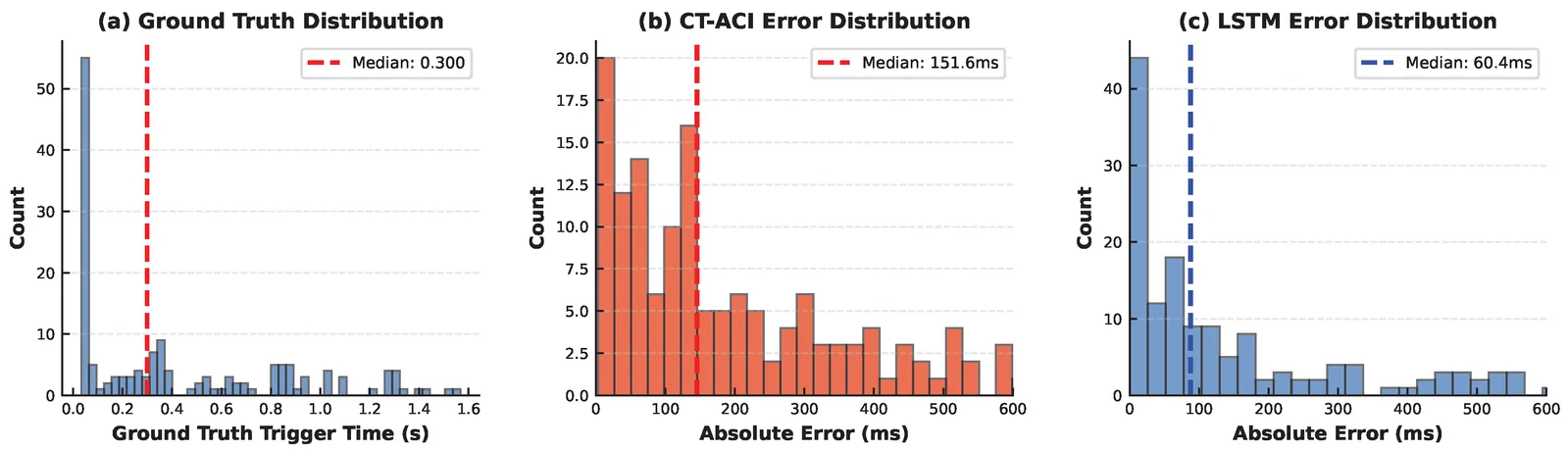
Distribution of absolute trigger-time prediction errors on the test set. CT-ACI exhibits a longer right tail (mean = 251.3 ms, SD = 264.4 ms, median = 151.6 ms, max = 1195.5 ms) compared with LSTM (mean = 205.3 ms, SD = 266.8 ms, median = 60.4 ms, max = 1291.5 ms).

**Figure 8:**
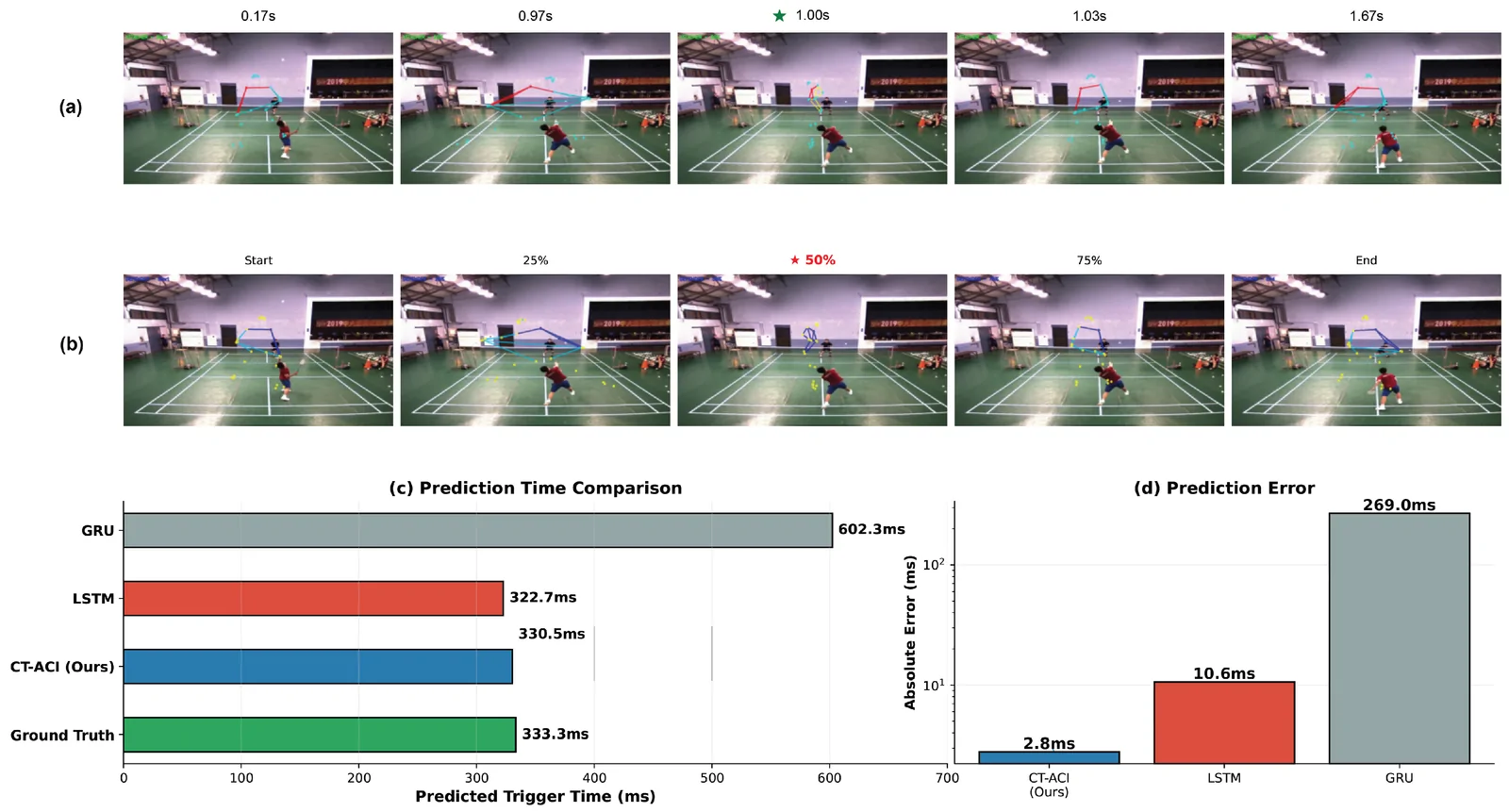
Representative long-rally case with a ground-truth trigger time of 333.3 ms after rally onset, corresponding to approximately 1.00 s on the video timeline. (**a**) The upper row shows five quality-filtered skeleton frames at the indicated video timestamps. The green star marks the ground-truth trigger time. (**b**) The lower row shows the corresponding rally positions normalized from the start to the end of the rally; the red star marks the 50% rally-progress position. In panels (**c**,**d**), the blue, red, and gray bars correspond to CT-ACI, LSTM, and GRU, respectively; in panel (**c**), the green bar indicates the ground-truth trigger time. CT-ACI, LSTM, and GRU predict trigger times of 330.5, 322.7, and 602.3 ms, respectively, corresponding to absolute errors of 2.8, 10.6, and 269.0 ms. CT-ACI achieves the lowest absolute error, approximately 3.8 times lower than LSTM and 96 times lower than GRU.

**Figure 9:**
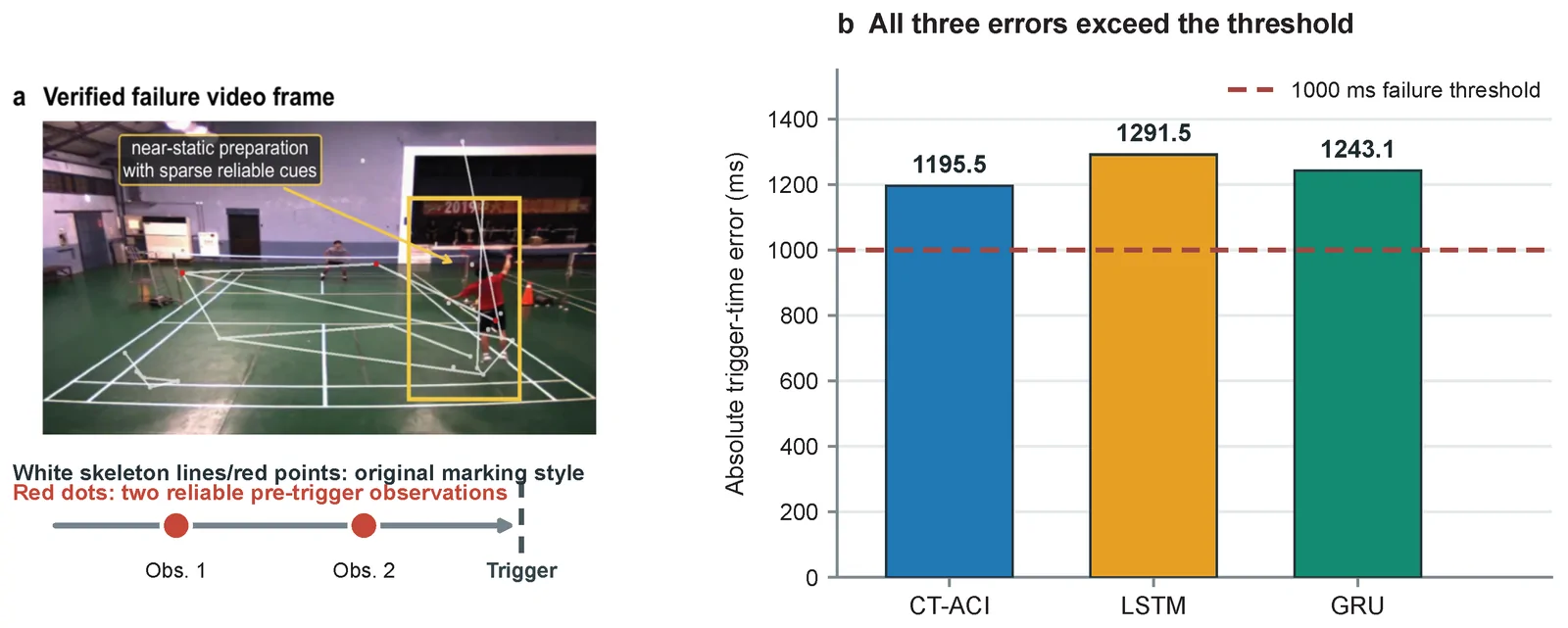
Failure-mode qualitative case. Only two reliable pre-trigger observations were available, and the attacker showed near-static preparation with sparse reliable cues. In panel (**a**), the red dots mark the two reliable pre-trigger observations (Obs. 1 and Obs. 2), and the dashed vertical line indicates the trigger point. In panel (**b**), the red dashed line indicates the 1000 ms failure threshold. All three methods exceed this threshold, with absolute trigger-time errors of 1195.5 ms for CT-ACI, 1291.5 ms for LSTM, and 1243.1 ms for GRU. CT-ACI, LSTM, and GRU all produce large trigger-time errors.

**Table 1:** TTE degradation at 90% missing frames across methods. All values are reported as median ± standard deviation across three independent runs. The comparison separates the contributions of the neural ODE dynamics and the temporal–causal attention mechanism.

Method	0% Missing (ms)	90% Missing (ms)	Degradation (%)
CT-ACI (Neural ODE + Attention)	151.6±4.23	198.5±6.14	30.9%
Standard ODE (Neural ODE only)	168.3±5.24	247.1±6.79	46.8%
LSTM (Discrete)	60.4±3.84	268.2±8.27	344.0%
GRU (Discrete)	61.8±4.07	325.2±9.68	426.2%

**Table 2:** Layer-wise ADE and FDE comparison between CT-ACI and LSTM. All values are reported as median ± standard deviation across three independent runs, in scaled normalized image-coordinate units (multiplied by 100 for numerical convenience). These values are meaningful only for relative comparison, and are not stated in centimeters.

Method	L1		L2		L3		L4	
	ADE	FDE	ADE	FDE	ADE	FDE	ADE	FDE
CT-ACI (Ours)	5.1±0.31	3.5±0.28	6.5±0.33	4.3±0.29	6.9±0.42	4.7±0.38	7.6±0.51	5.4±0.47
LSTM	3.7±0.22	2.4±0.19	5.3±0.31	3.8±0.27	7.4±0.43	5.2±0.41	9.0±0.62	8.0±0.68

**Table 3:** Per-method match-level sensitivity on the test set. ΔTTE and ΔAccuracy denote test-holdout minus test-overlap. Relative changes are computed with respect to the test-overlap subset. Lower ΔTTE indicates less timing degradation on the holdout match. Negative ΔAccuracy indicates an accuracy drop on the holdout match.

Method	ΔTTE (ms)	ΔTTE (%)	ΔAccuracy (pp)	ΔAccuracy (%)
CT-ACI	+14.0	+9.5	−1.7	−2.1
LSTM	+7.0	+12.1	−2.0	−2.6

**Table 4:** Numerical ablation results on the test set. TTE values are reported as median with interquartile range (Q1, Q3). Accuracy values are reported as mean with 95% confidence interval (lower, upper). Bold indicates the best performance for each metric. ↓ indicates that lower values are better, and ↑ indicates that higher values are better.

Model Variant	TTE (ms) ↓	Accuracy (%) ↑
Full CT-ACI	**151.6 [61.7–361.0]**	**78.7 [77.5–79.9]**
w/o Neural ODE (GRU replacement)	164.3 [95.5–326.6]	74.1 [72.0–76.2]
w/o Adaptive Threshold	156.8 [64.0–395.8]	76.3 [75.0–77.6]
w/o Causal Attention	170.8 [62.0–405.0]	76.7 [75.4–78.0]
w/o Curriculum Learning	153.9 [51.1–382.9]	75.5 [74.2–76.8]

**Table 5:** Sensitivity of median TTE to varying primary threshold θ (NVU) on the test set. The model predictions are fixed (trained with θ=0.3); labels are regenerated for each θ.

θ (NVU)	Median TTE (ms)	IQR [Q1, Q3] (ms)	Δ vs. θ=0.3
0.10	258.4	[142.3, 489.1]	+70.5%
0.15	218.7	[118.5, 425.3]	+44.3%
0.20	183.2	[92.4, 398.7]	+20.8%
0.25	162.5	[75.8, 380.2]	+7.2%
0.30	151.6	[61.7, 361.0]	—
0.35	155.8	[63.5, 372.4]	+2.8%
0.40	179.3	[80.1, 401.5]	+18.3%
0.45	215.6	[110.3, 438.7]	+42.2%
0.50	262.1	[145.0, 497.2]	+72.9%

**Table 6:** Joint-specific confidence and failure statistics for the hip landmarks across all parsed frames from the training, validation, and test splits. Hip-center confidence is defined as the minimum of the left and right hip confidences, following a conservative criterion that a frame is reliable only when both hip landmarks are reliably detected. The failure rate is the fraction of frames for which confidence falls below the threshold of 0.5.

Landmark	Mean	SD	Median	5th Percentile	Failure Rate (%)
Left hip	0.89	0.08	0.92	0.71	1.8
Right hip	0.88	0.09	0.91	0.69	2.1
Hip center (min)	0.87	0.09	0.91	0.62	2.3

**Table 7:** Comparison of representative 2D pose estimation frameworks in terms of hip-center localization error, CPU inference speed, hardware requirement, and deployment characteristics. Hip-center error is computed on a stratified subset of 100 test clips using ViTPose-B as the reference and is reported as the mean normalized Euclidean distance. ViTPose-B is used as a practical reference because it achieves the highest reported COCO AP among the compared frameworks and its error is defined as zero by construction. Lower error indicates higher accuracy. CPU speeds depend on hardware and input resolution.

Framework	Hip-Center Error (Normalized)	CPU Speed	Hardware	Deployment
MediaPipe Pose	0.025	15–25 ms/frame	CPU only	Lightweight, cross-platform
OpenPose	0.022	80–200 ms/frame	GPU recommended	Multi-person, resource-heavy
MoveNet	0.028	10–20 ms/frame	CPU/Edge TPU	Mobile-optimized
AlphaPose	0.018	GPU-dependent	GPU required	Occlusion-robust
RTMPose-m	0.012	90+ FPS (i7-11700)	CPU friendly	Real-time multi-person
ViTPose-B	0.000 reference	GPU-dependent	GPU required	Highest accuracy

**Table 8:** Stratified consistency of $T^{*}$ labels between MediaPipe and each alternative pose estimator across difficulty layers. Values are Pearson correlations ρ computed on the stratified random subset described in the text.

Estimator	Layer 1	Layer 2	Layer 3	Layer 4
ViTPose	0.995	0.993	0.990	0.985
RTMPose	0.992	0.989	0.985	0.978
AlphaPose	0.988	0.985	0.980	0.972
OpenPose	0.984	0.980	0.974	0.964
MoveNet	0.978	0.974	0.967	0.956

**Table 9:** Unified performance summary on the test set under 0% and 90% missing frames. All values are reported as median ± standard deviation across three independent runs. ADE and FDE are reported in scaled normalized image coordinate units (multiplied by 100 for numerical convenience). Bold indicates the best performance for each metric. ↓ indicates that lower values are better, and ↑ indicates that higher values are better. ^†^ denotes the adapted SkelVIT baseline.

Missing Ratio	Method	TTE (ms) ↓	Accuracy (%) ↑	ADE (Scaled Units) ↓	FDE (Scaled Units) ↓
0%	CT-ACI (Ours)	151.6±4.23	**78.7 ± 0.58**	6.3±0.32	4.2±0.41
	LSTM	60.4±3.84	76.1±0.82	5.1±0.23	3.4±0.29
	GRU	61.8±4.07	75.9±0.88	5.6±0.31	4.7±0.37
	Standard ODE	168.3±5.24	70.6±1.13	6.8±0.42	5.6±0.48
	Causal GCN	156.0±4.53	72.8±0.97	6.6±0.34	5.3±0.52
	SkelVIT (adapted) ^†^	155.8±4.76	76.4±0.91	7.9±0.43	6.6±0.57
90%	CT-ACI (Ours)	**198.5 ± 6.14**	**72.6 ± 0.79**	7.2±0.52	6.5±0.51
	LSTM	268.2±8.27	61.5±1.24	10.8±0.83	7.9±0.77
	GRU	325.2±9.68	59.0±1.38	12.6±1.02	10.8±0.96
	Standard ODE	247.1±6.79	65.2±1.08	9.1±0.63	7.4±0.72
	Causal GCN	216.3±5.87	64.0±1.04	9.5±0.69	7.8±0.73
	SkelVIT (adapted) ^†^	285.0±7.42	67.3±1.01	13.2±0.92	11.0±1.03

**Table 10:** Paired Wilcoxon signed-rank tests between CT-ACI and each baseline on overall accuracy under the standard test condition. Effect sizes are reported as rank-biserial correlations. All tests are two-sided at the 0.05 significance level.

Baseline	*p*-Value	Rank-Biserial *r*
LSTM	0.012	0.08
GRU	0.021	0.12
Standard ODE	<0.001	0.28
Causal GCN	<0.001	0.21
SkelVIT (adapted)	0.034	0.09

**Table 11:** Trigger-time error distribution statistics on the test set. All values are in milliseconds.

Method	Mean	SD	Median	Max
CT-ACI (Ours)	251.3	264.4	151.6	1195.5
LSTM	205.3	266.8	60.4	1291.5

## Data Availability

The VideoBadminton dataset [33] is publicly available. The download link is: https://drive.google.com/file/d/1KW5Q8Hd8-J75FdnehMjHw1WvdXDv1UxB/view (accessed on 3 April 2026). The processed data generated and analyzed in this study, as well as the analysis code, are available from the corresponding author upon reasonable request.
